# Individual differences and motor planning influence self-recognition of actions

**DOI:** 10.1371/journal.pone.0303820

**Published:** 2024-07-30

**Authors:** Akila Kadambi, Qi Xie, Hongjing Lu

**Affiliations:** 1 Department of Psychology, UCLA, Los Angeles, California, United States of America; 2 Department of Statistics, UCLA, Los Angeles, California, United States of America; Tokai University, JAPAN

## Abstract

Although humans can recognize their body movements in point-light displays, self-recognition ability varies substantially across action types and participants. Are these variations primarily due to an awareness of visually distinct movement patterns, or to underlying factors related to motoric planning and/or individual differences? To address this question, we conducted a large-scale study in self-action recognition (*N* = 101). We motion captured whole-body movements of participants who performed 27 different actions across action goals and degree of motor planning. After a long delay period (~ 1 month), participants were tested in a self-recognition task: identifying their point-light action amongst three other point-light actors performing identical actions. We report a self-advantage effect from point-light actions, consistent with prior work in self-action recognition. Further, we found that self-recognition was modulated by the action complexity (associated with the degree of motor planning in performed actions) and individual differences linked to motor imagery and subclinical autism and schizotypy. Using dynamic time warping, we found sparse evidence in support of visual distinctiveness as a primary contributor to self-recognition, though speed distinctiveness negatively influenced self-recognition performance. Together, our results reveal that self-action recognition involves more than an awareness of visually distinct movements, with important implications for how the motor system may be involved.

## Introduction

Recognition of oneself depends on more than visual experience. As a prime example, humans can recognize their own actions even from minimal visual input provided by point-light action displays [1–3]—disconnected dot animations depicting the motion of key joints on the human body [4]. Despite removing a large degree of body identity information and depicting actions in unfamiliar, third-person perspectives, self-recognition from point-light movements suggests that people can readily connect motor experience of performing actions to visual recognition of actions. These findings are consistent with multiple lines of evidence that have revealed close links between motor and visual action representations [e.g., 5–11].

Previous research has identified a few core characteristics of self-recognition from actions. From point-light displays, participants are more accurate in recognizing themselves than visually familiar friends [1], even when configural topographic cues are disrupted [12, cf. 2], or visual feedback is removed [9, 13]. People can identify their own actions from sparse whole-body movements [2] and even from isolated body parts, such as hand movements [14]. Self-recognition of one’s own gait is viewpoint-invariant [cf. 15] but recognition of familiar friends from their gait patterns is viewpoint-dependent, with better performance from frontal than profile views [16]. View-independent performance for self-recognition from point-lights has also been observed for actions less common than walking [2, 17] such as dancing or jumping [18], suggesting that the visual representation of self-actions based primarily on motor experience is fundamentally different from action representations based on visual experience of others. The self-action advantage also extends beyond explicit visual recognition of whole bodies, showing greater predictive accuracy of future action outcomes [e.g., 8, 19, cf. 20], identity-irrelevant (implicit) body part recognition [21–23], facial expression recognition [12], memory for self-performed action verbs (i.e., enactment) [24], and action recognition across multiple modalities [e.g., 25–28].

While these studies suggest that self-action recognition can arise from prior motor experience despite lacking copious visual experience, the underlying mechanisms are yet to be clarified. On the one hand, it is possible that self-recognition from our actions could be primarily driven by a visual awareness of one’s own movement style or noticing the visual distinctiveness of certain movements. A recent study by [29] showed that self-recognition performance was based on the degree of movement similarity conveyed by self-generated actions relative to actions performed by others. In this study, participants were asked to perform postural motions with general instructions such as “*create postural motions by keeping knees extended with toes and heels in constant contact with the floor*.” Although people recognized their own actions above chance, people more often misattributed movements of others to themselves that were highly visually similar. These results suggest that people recognize themselves in point-light displays based on the degree of visual similarity to their own kinematic styles of body movements. Notably, however, the study gave participants the same general instructions in action performance without varying the action goal or complexity. When the goal complexity of our own actions and actions performed by others is manipulated, other potential mechanisms could further influence self-recognition.

Prior work has shown that the degree of motor planning required to produce an action modulates the motoric goal complexity of the action, which often produces the visual distinctiveness in the action sequence. That is, action goals with more complexity require more motor planning than actions with simple goals. Complex actions also evoke different neural activity in brain networks related to action observation, even when the same effectors are used [30, 31]. Further, self-recognition performance varies substantially across different actions that vary in their goal complexity. [2] recorded ten actions from each individual participant and asked participants to report the identity (self, friend or stranger) of the point-light actors. The researchers found that identity performance varied significantly across actions in the range of 40~80% (chance level of 33.3%). For example, people were more accurate in self-recognition for complex actions such as dancing (~80%) and boxing (~65%), than for relatively simple and routine actions such as walking/running (~40%). Similarly, [32] found greater self-recognition performance for dancing movements than for stereotyped gait patterns. Thus, the identification of self-actions can be affected by action-level variance in goal complexity, driven internally by the amount of motor planning required during action production.

Another important influence on performance variability in self-recognition is participant-level variance. Individual differences in motor imagery influence performance on biological motion tasks [e.g., 33], which may further impact how people recognize their own biological motion. Moreover, motor imagery is part of a larger action simulation network, which can be evoked either through action observation of others or through self-imaging [e.g., 34]. Action recognition and motor simulation are also well-characterized in the literature as sharing partially overlapping neural and behavioral resources [35–37]. Thus, it is possible that individuals with more motor imagery ability may be better able to recognize their own actions when viewing them in point-light displays.

Another correlate of participant variance could be linked to clinical variability in sensorimotor processing. Sensorimotor self-processing difficulties are notably characterized in the Autism Spectrum Conditions (ASC)–from the Greek root “autos” for self [38–45] and Schizophrenia Spectrum Conditions (SSC), a “disordered self”, characterized by “a disunity of consciousness” [46–51]. In these conditions, altered self-representations are present and attributed to an early disturbance at the bodily level [40, 51–55]. In ASC, studies show sharpened sensorimotor boundaries between the self and other [52, 56], while in SSC the boundary appears to be weakened or blurred [51, 52, 57–60], though see different directionality in [61, 62]. Trait variability in the general population linked to ASC further impacts self-action recognition from point-light displays [17], bodily self-awareness [56] and is linked to alterations in one’s self-concept and autobiographical reasoning ability [63]. Trait variability linked to SSC impacts the sense of agency [64, 65], spatial self-boundaries [61, 62], self-face recognition [66], and sensorimotor outcome prediction [67]. Further, trait variability in both conditions is linked to atypical processing of human actions [33, 65, 67–72]. Thus in addition to the influence of motor planning, self-recognition performance may further be impacted by individual differences in autism-spectrum and schizotypal traits, relevant to sensorimotor self-processing difficulties.

To assess the primary factors underlying performance differences in self-action recognition, we conducted the first large-scale study (*N* = 101) in self-action recognition and asked the following: does self-recognition depend on systematic properties from motoric factors of the actions and/or individual differences of the participants? How does the visual distinctiveness of movements impact self-recognition when the goal complexity of the action is manipulated? We varied the degree of motor planning involved in the action type, by including actions from three categories: *simple* actions (simple goals, less motor planning), *complex* actions (complex goals, more motor planning), and *imitation* actions (least motor planning based on copying another person’s motor plan) [73]. These actions were primarily selected from [17] who previously modified the goal complexity of the actions based on the degree of motor planning needed to perform the action. For the individual difference measures, we included vividness of motor imagery, autism-spectrum, and schizotypal traits. Finally, we measured the contribution of visual distinctiveness of movements on self-action recognition using a widely-used algorithm in spatiotemporal signal processing—dynamic time warping (DTW) [74]—to quantify action similarity between self-movements and actions performed by others based on movement trajectories of joints in actions.

The experimental design consisted of two sessions. In the first session, 27 actions were performed by each participant and recorded through motion capture. Participants were informed that their actions were recorded for an action recognition study but were never informed of the study’s focus on self-recognition. After a delay of about one month on average, participants returned to complete a self-recognition task that required recognizing their own action among three other actors performing the same action. We introduced three types of actions during the motion capture session: nine simple actions and nine complex actions that were verbally instructed (i.e., “please naturalistically perform the action: *to grab / to get attention*”), and nine actions that were provided with video instruction, asking participants to imitate body movements of an actor shown in a video. We included video instruction since imitation elicits a unique action requirement: copying another’s motor sequence, thereby reducing motor planning demands of spontaneous action production (see enactment effect) [73]. If self-recognition performance depends on the degree of naturalistic motor planning involved during action production, we would expect that imitation actions may yield lower accuracy in identifying own body movements than complex actions provided with verbal labels, even at comparable levels of action complexity and across large variability in performing the same actions across individuals.

Therefore, we hypothesized that participants would not only recognize their own actions in point-light displays, but that recognition performance would vary systematically, according to underlying differences in motor planning and intrinsic traits of participants. We further hypothesized that actions that required more motor planning during action production would lead to greater visual self-recognition relative to actions with less motor planning. Additionally, we expected that self-recognition performance should correlate negatively with profiles of autistic and schizotypal traits and correlate positively with motor imagery ability.

## Method

### Participants

108 undergraduate students (*M*_age_ = 21.20, *SD*_age_ = 3.81, females = 79, males = 29) were recruited through the Subject Pool at the University of California, Los Angeles. Sample size was determined in accord with another large-scale individual differences study examining the relation between autistic and schizotypal traits and emotion discrimination from biological movement patterns [75]. The study was approved by the UCLA Institutional Review board. All participants received course credit, which was granted as a recognition of their contribution to the research after completion of the study. All participants were naïve to the purpose of the study. Participants had normal or corrected-to-normal vision and no physical disabilities. Seven participants were excluded due to inputting errors of participant motion capture files, resulting in a total of 101 participants included in the analysis (M = 28, F = 73).

### Apparatus

**Fig 1:**
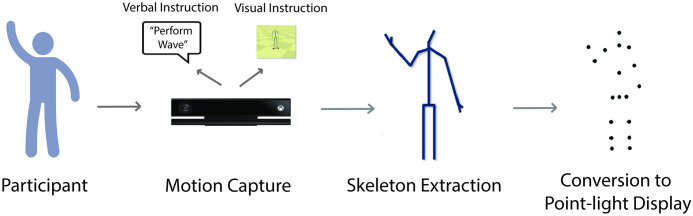
Illustration of motion recording (session 1). Actions were recorded by the Kinect system and converted to point-light displays. Participants were instructed to perform the actions either through verbal instruction (simple, complex) or visual instruction guided by a stick figure (imitation).

Participants’ body movements were recorded using the Microsoft Kinect V2.0 and Kinect SDK in a quiet testing room. Participants were instructed to perform the actions in a rectangular 0.76 m x 1.52 m space, in order to provide flexibility to perform the action, while remaining within recording distance. The Kinect was placed 1.52 m above the floor and 2.59 m away from the participant. The three-dimensional (X-Y-Z) coordinates of the key joints were extracted at a rate of approximately 33 frames per second and later used to generate point-light displays of actions (see Fig 1). Customized software developed in our lab was utilized to enhance movement signals, and to carry out additional processing and trimming for actions presented later in the testing phase [76].

### Stimuli generation

All actions were selected to be commonly encountered actions and were captured by the motion capture system. The actions varied in complexity in order to characterize a broad range of common movements in daily life. First, participants were instructed to freely performed nine simple and nine complex actions provided with clear verbal labels (i.e., verbal instruction). Participants indicated the start/stop of their action with a T-position. Simple and complex actions were extended from [17] delineated by whether the action was a simple goal (e.g., *wave*), conveying a routine action with less motor planning, or a complex goal (e.g., *argue*) linked to more detailed motor planning. The simple actions included *grab*, *jump*, *wave*, *lift*, *kick*, *hammer*, *push*, *point*, *punch*. The complex actions included: *argue*, *macarena*, *wash windows*, *play baseball*, *get attention*, *hurry up*, *fight*, *stretch*, and *play guitar*. Note that although we used the degree of involvement for motor planning to create the dichotomy of simple and complex actions, the two categories could differ in other dimensions. For example, some actions may show greater variability in speed when performed by different individuals than other actions.

In addition to performing actions instructed by verbal labels, participants also performed actions based on video instruction. Participants were asked to view an action performed by actors shown in a stick-figure display (i.e., lines connected between joint positions) on a computer, and subsequently imitate the seen action afterwards. Nine imitation actions were selected from the Carnegie Mellon Graphics (CMU) Lab Motion Capture Database available online (http://mocap.cs.cmu.edu), generated from predefined actors. The actions were selected to capture a broad range of goal variability: *jumping jacks*, *playing basketball*, *bend*, *direct traffic 1*, *direct traffic 2*, *conversation*, *laugh*, *digging a hole*, *and chopping wood*. To account for any implicit goal-attribution or familiarity with action sequences that could impact the degree and/or type of imitation [77–79], we included a range of familiar (e.g., bend) and unfamiliar (e.g., directing traffic) imitation action sequences. Participants were never provided the verbal label for imitation actions. Each video displayed an actor shown as a stick figure performing one of the imitation actions and was presented in three different angles to the subject, either to the right or left (+/− 45°; half-profile) or facing forward (0°; frontal) by rotating the horizontal axis. Each imitation action was recorded twice: once after viewing the three different angles, and once more after viewing only the forward-facing angle. The first imitation recording was discarded (served as practice during motion recording), and only the second imitation recording was used in the self-recognition experiment. The recorded raw motion data from the Kinect system were passed through a double exponential adaptive smoothing filter [80] to remove noisy and jittered movements (e.g., ballistic random jumps of points). Additionally, the stimuli were trimmed and processed to display the point light-displays using BioMotion Toolbox [76] with their segmented action recording, which would be reiteratively looped in the self-recognition session.

### Procedure

The experiment was split into two sessions: motion recording and recognition testing. The first session consisted of a motion recording session, where participants performed the actions and were recorded with a motion capture system. Participants were informed that their actions were recorded for an action recognition study, but were never informed about the aim of the study. After a delay period (*M* = 37.39 days, *SD* = 5.20 days; range: 23–56 days), participants returned for the second session to complete two recognition tasks. In one of the tasks from the recognition session, participants underwent a self-recognition task by identifying their own actions that were recorded with the motion capture system. In the other task, participants completed a visual recognition task, where they identified actions that they imitated during the motion recording session (further detailed below). The order of the self-recognition task and the visual recognition task was counterbalanced between participants.

#### Session 1: Motion capture

During the action recording in Session 1, participants were provided verbal instruction for the 18 actions (nine simple and nine complex) and asked to perform the actions as naturally as possible (i.e., “please naturalistically perform the action: *to grab*”). For the remaining nine imitation actions, none of the participants were provided the verbal label of the action. Instead, participants were instructed to *imitate* the movements of the action presented in a stick figure video (Fig 1).

Upon completing the action recording, participants completed two questionnaires: Schizotypal Personality Questionnaire (SPQ) [81] and the revised Vividness of Motor Imagery Questionnaire (VMIQ-2) [82]. The SPQ was administered to assess degrees of schizotypal traits among individuals in the typical population. The VMIQ-2 was included to assess motor imagery differences as a potential source of variability in self-processing and biological motion processing.

#### Session 2: Self-Recognition task and visual-recognition task

**Fig 2:**
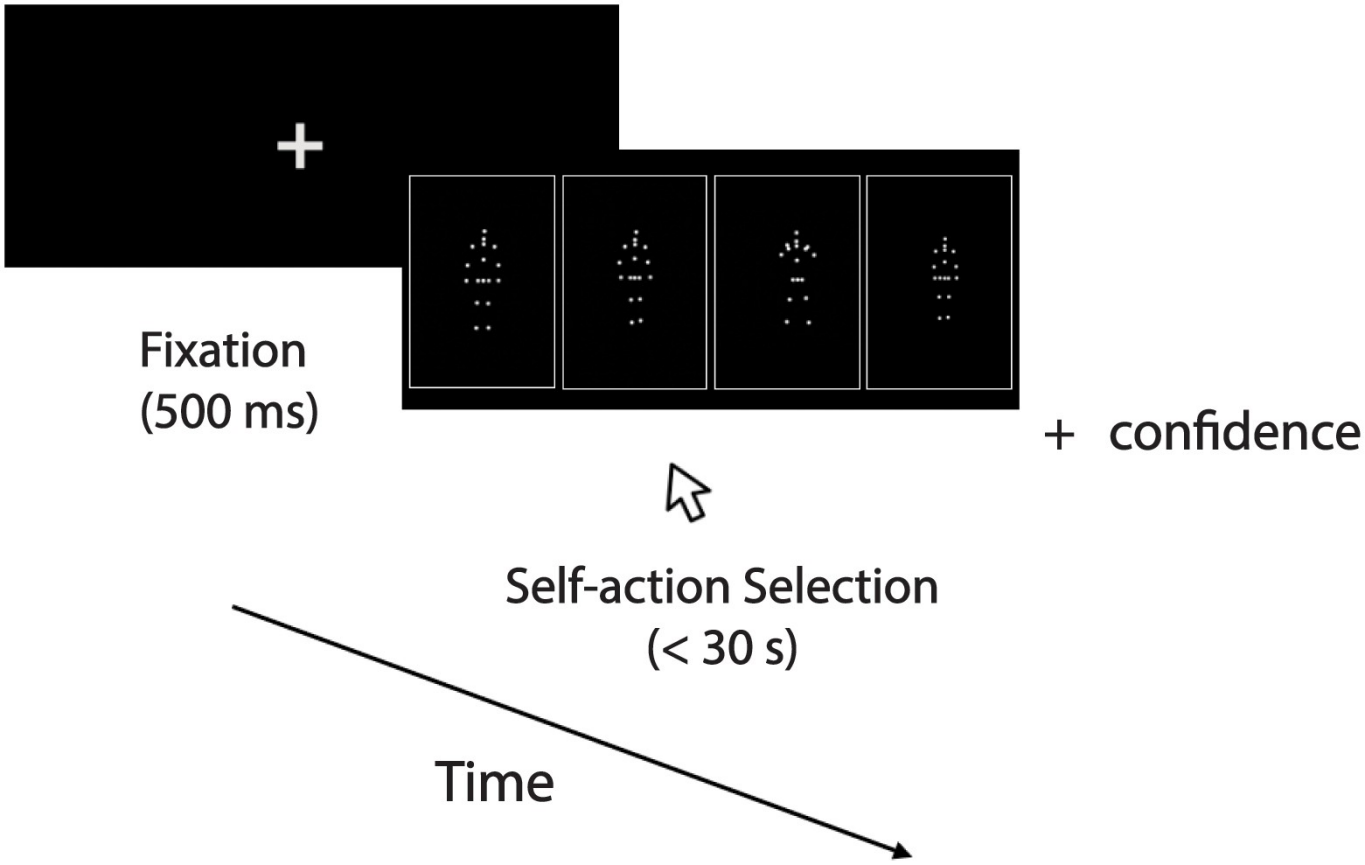
Schematic illustration of a sample trial showing “push” action for the self-recognition task. One point-light action is the participant’s action, while the other three point-light actions are distractors performed by sex-matched actors. During the display of actions, participants were provided a maximum of 30 seconds to click on the action that was their own. Following the actions, participants rated their overall confidence in the selection (1: not at all confident to 5: most confident).

In the second phase (recognition test; Fig 2), participants were seated approximately 0.76 meters in front of a monitor in a dimly lit room and were asked to select their own action amongst three other distractor actions spread out horizontally along the center of the screen, as shown in Fig 2. Each action was presented with 17 point-lights located at key joints, in three different orientations (rotated around the vertical axis) 0°, (frontal), 45° (half-profile, right), 225° (half-profile, left), for a total of 81 trials. All of the actions within a trial displayed the same orientation. Participants were instructed to select their own point-light action amongst four displays. The actions were looped until the participant selected one of the point-light actors, each depicted in one of four horizontally-spread, randomly arranged boxes, or until a time limit of 30 seconds. The four animations included their own action and the same action performed by three distractor actors with the same sex, all of whom participants were unfamiliar with. All the point-light actions were normalized for maximum height. Following selection of the action, participants were asked to provide a confidence judgment, in which they rated the confidence of their selection from 1 (not at all confident) to 5 (most confident). Participants were not provided any feedback.

The first 28 participants only underwent the self-recognition task. The subsequent 73 participants were tested with both the self-recognition task and the additional visual recognition task. The visual recognition task consisted of nine trials depicting the forward-facing imitation actions. In this task, participants were instructed to identify the actor they imitated during the motion recording phase (converted to point-light display) amongst three other distractor point-light actors who performed the same action. That is, the actor they were instructed to identify in this task was the same as the actor they imitated in Session 1. The distractor actors were identical to those used in the self-recognition task. The visual recognition task measured recognition performance of imitated actions that participant had previously seen and were performed by other actors. Thus, by comparing performance on the visual recognition task to performance on the self-recognition task, we could assess the separate contribution of motor experience (that included motor planning and motor familiarity) from the contribution of visual experience to recognition performance. The visual recognition trials used the identical stimulus layout as in the self-recognition task, except that the participants’ own action in the self-recognition trials was replaced by the point-light actions of original imitation actor from the CMU motion capture database. The order of presentation of the visual recognition task was counterbalanced to either follow or precede the self-recognition task across participants. Following both recognition tasks, participants were asked to complete the Autism-Spectrum Quotient (AQ) questionnaire to assess their degree of Autistic traits [83].

### Individual difference measures

#### Autistic quotient

We assessed self-reported autism-spectrum traits in the general population using the Autism-Spectrum Quotient (AQ) questionnaire, consisting of 50 questions designed to measure five different subtypes: *social skill*, *attention switching*, *attention to detail*, *social communication*, and *imagination* [83]. Response criteria requires the selection of one of the four possibilities (four-point scale): “definitely disagree”, “slightly disagree”, “slightly agree”, “definitely agree.” While not a diagnostic instrument, scores of 32+ on the AQ in the general population are generally indicative of a predisposition to ASC, out of a maximum score of 50 points (1 point per question validating autism-spectrum traits), while the cutoff for ASC individuals on the AQ is typically greater than 26 [84, 85].

#### Schizotypal personality questionnaire

To measure trait-variance related to Schizotypal Personality Disorder amongst neurotypicals, we used the Schizotypal Personality Questionnaire (SPQ) developed by [81]. The 74-item survey was based on criteria from the *DSM-III-R* [86] that measures schizotypy from multiple dimensions (positive, negative, disorganized, and paranoia) and captures its phenotype, etiology, symptomatology [87, 88]. We chose the full-scale SPQ (74 items) rather than the more recent SPQ-brief (32 items) [89] as the original SPQ has been shown to provide a clearer division of the individual subscales [90] important when considering the reflected overlap between AQ and SPQ subscales. The SPQ adopts a three-factor structure (analogous to the symptom structure in Schizotypal Personality Disorder and SSC), measuring three main constructs of schizotypy: the cognitive-perceptual dimension (positive schizotypy), interpersonal dimension (negative schizotypy), and disorganized feature dimension (disorganized schizotypy) based on *DSM-III-R* criteria [91] but well-matched to current *DSM-V* criteria [88]. Further divisions within the three-factor structure include nine different subscales of the SPQ: *ideas of reference*, *unusual perceptual experiences*, *odd/magical beliefs*, *suspiciousness/paranoid ideation* (cognitive-perceptual); *social anxiety*, *no close friends*, *constricted affect* (interpersonal); *odd behavior and appearance*, and *odd speech* (disorganized) [81, 91]. Response criteria on the questionnaire requires binary selection of “true” or “false” (two-point scale) to particular statements (e.g., “I am aware that people notice me when I go out for a meal or to see a film”). The top 10% of scorers typically reflect scores greater than 41, while the bottom 10% typically score 12 or lower.

#### Vividness of Motor Imagery Questionnaire

The VMIQ-2 [82] measures introspective reports of vividness of imagery in kinesthetic (movement simulation), internal (first person simulation), and external (third person simulation) visual imagery of 12 different actions (e.g., kicking). Vividness of motor imagery is rated on a five-point Likert scale for each of the 12 actions in each of the three sub-areas. Note that lower scores in VMIQ-2 indicate more vivid images and stronger motor imagery ability.

### Action similarity measures

#### Movement distinctiveness (dynamic time warping)

**Fig 3:**
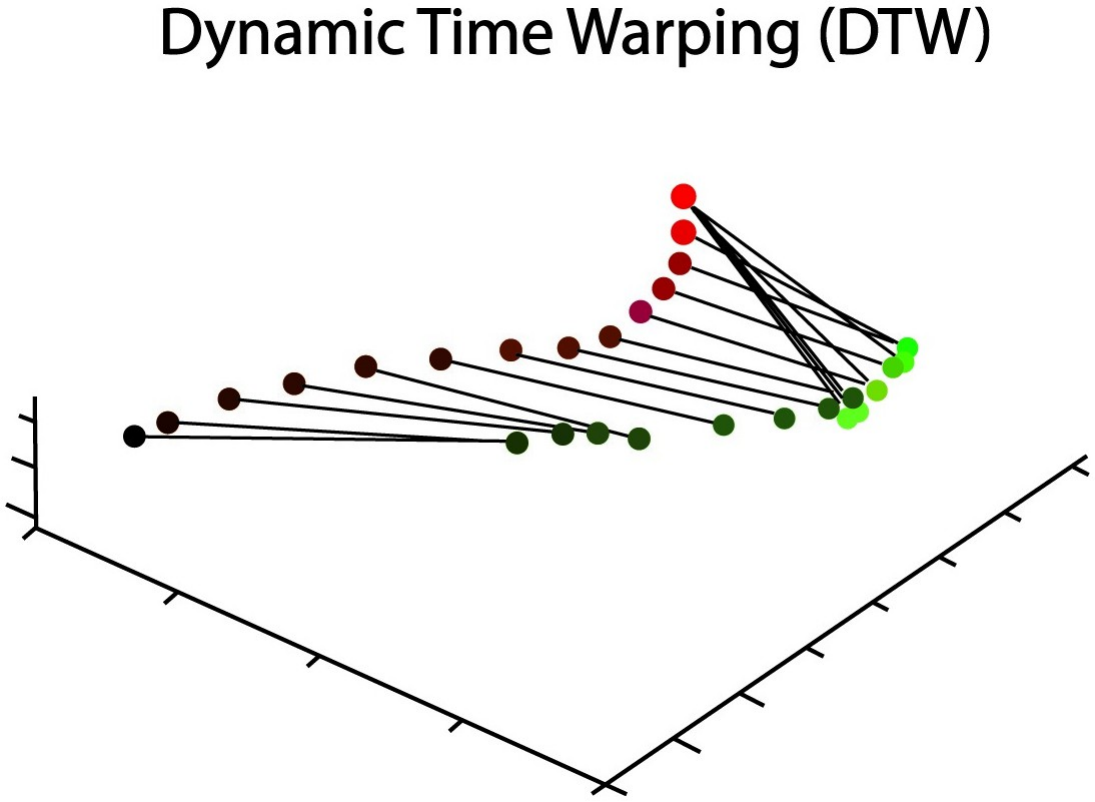
Illustration of DTW alignment via temporal warping for joint movements (sample joint: Elbow) in an action (sample action: Wave) between performed by two actors (red or green). The gradient in circle colors indicates the elapse of time, with darker colors reflective of earlier frames, and brighter color in later frames. The temporal warping function is illustrated as connected lines.

People perform some actions with highly similar movements across individuals, but other actions with distinctively different body movements. To assess the contribution of movement distinctiveness to self-recognition, we implemented the dynamic time warping (DTW) algorithm (visualized in Fig 3).

DTW provides a quantitative measure of similarity between temporal sequences using a nonlinear, monotonic temporal warping algorithm [74]. The DTW algorithm is designed to find an optimal match (known as a warping path) for temporal alignment between pairwise time sequences that minimizes their dissimilarity regardless of low-level factors (e.g., speed, duration differences). Smaller DTW distance values indicate greater similarity of body movements between actors when they performed the same action based on motion trajectories in actions. DTW values were computed based on task demands during the self-recognition task, in which participants compared their own action to three other distractor actions. This was computed as the minimum distance of the participant’s action relative to all distractors’ actions shown in the task.

The following steps were implemented for DTW analyses in MATLAB R2020A. A plain language implementation (pseudo-code) of the DTW algorithm can also be found in [92].

1. For each participant’s actions, we extracted the 3D positions of each of the 17 joints using the BioMotion toolbox [76].
2. We centered each trajectory of a joint to zero in order to remove the impact of global factors (e.g., global body displacements, limb length, etc.) on the similarity measures.
3. We implemented an action DTW algorithm [92] to search for a temporal warping function shared across all 17 joints. See Fig 3 for the illustration of temporal warping for joint trajectories performed by two actors in the action DTW algorithm.
4. After deriving the optimal warping function, the analysis computes the frame-by-frame Euclidean distances of the temporally warped joint trajectories in actions performed by different actors.
5. DTW distance was then computed as the sum of the distances between all joint trajectories normalized by the number of frames of a target actor. This normalization step is required in order to account for the different durations across participants performing the same action
6. For each participant, the dissimilarity of the target participant performing an action from the three distractor actors in the experiment was captured by a minimum DTW distance measure, computed by capturing the minimum pairwise DTW distance between the target participant across each distractor in performing this action. We chose to use the minimum distance since distinctiveness during the task relies on a relative comparison to each actor shown in the trial, rather than averaging across all three distractors.

#### Action duration and speed

In addition to movement dissimilarity, people may also perform actions with different durations or speed of movements. Since these visual cues are not analyzed in the DTW algorithm, we further computed action metrics related to distinctiveness in speed and duration, and measured their influence on self-recognition performance. Action duration was computed as the amount of time used to perform the same action by different individuals. Action speed used the 3D joint positions to calculate the mean speed, which was averaged across time and all 17 joints. Differences in speed and duration were then computed by taking the minimum difference between the averaged absolute speed/duration differences between the action performed by a target participant relative to all the distractor actions. For each feature, we measured pairwise distances between each action performed by the participant and the actions performed by the other individuals and distractor actions. The minimum pairwise feature distance was then taken to capture one overall feature dissimilarity for the action of the participant relative to all distractors and the average pairwise distance was taken relative to all participants.

The following steps were implemented for both speed and action duration analyses in MATLAB R2020A:

1. For each participant’s actions, we extracted the 3D positions of each of the 17 joints using the BioMotion toolbox [76].
2. Each trajectory of a joint was centered to zero in order to remove the impact of global factors (e.g., global body displacements, limb length, etc.) on the similarity measures.
3. For speed, differences were computed between each action extracted from 3D joint positions based on their Euclidean distance.
4. For each participant, the dissimilarity of the target participant performing an action from the three distractor actors in the experiment was captured by a mean duration/speed distance measure, computed by averaging across pairwise duration/speed distances between the target participant with each distractor in performing this action. In addition, we also used the same algorithm to compute the dissimilarity of the target participants performing an action from all other participants.

## Results

### Impact of extrinsic factors (action types) on self-recognition performance

All analyses were implemented using SPSS 25.0 (IBM SPSS, Armonk, NY), R (Version 4.2.1), and MATLAB R2020A. Linear mixed modeling was implemented using the lme4 [93] and lmerTest [94] packages in R and significance of fixed effects was estimated using Satterthwaite’s approximation for degrees of freedom of F statistics. Planned post-hoc comparisons for the mixed model used the function emmeans() in R [95] with degrees of freedom adjusted using Kenward-Roger approximation. Models were estimated using restricted maximum likelihood.

**Fig 4:**
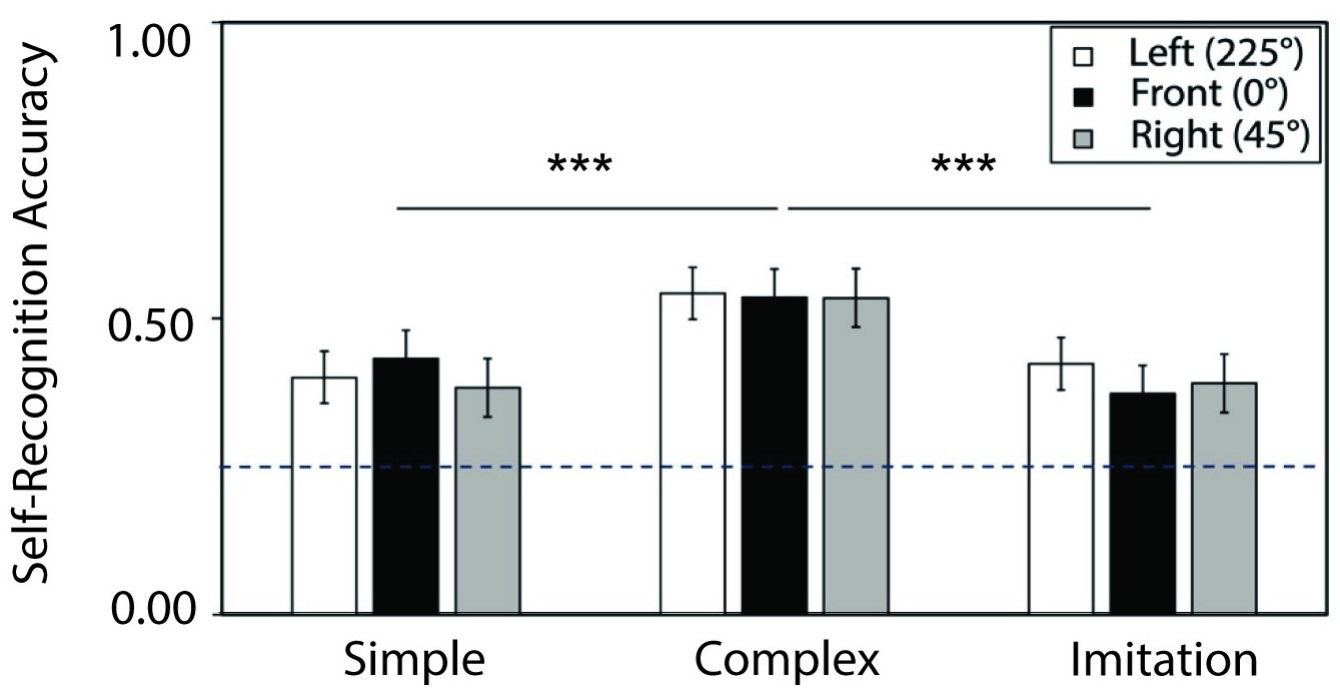
Results of self-recognition accuracy by action types (x-axis) and orientation (colored bars). All action types recognized significantly above chance regardless of viewpoint. Complex actions showed greater self-recognition performance than did simple or imitation actions. Dashed line indicates chance performance (0.25). The error bars indicate standard error of means.

Shown in Fig 4, participants were able to recognize self-actions significantly above chance performance (0.25 in a 4 alternative-forced-choice task): for simple actions with verbal instruction (*M* = 0.40, *SD* = 0.16), *t*(100) = 9.45, *p* < .001, cohen’s *d* = .940, for complex actions with verbal instruction (*M* = 0.54, *SD* = 0.17), *t*(100) = 16.86, *p* < .001, *d* = 1.678, and for imitated actions without verbal instruction (*M* = 0.39, *SD* = 0.16), *t*(100) = 9.20, *p* < .001, *d* = .916, corroborating prior research on self-action recognition from point-light displays [e.g., 2, 17]. Confidence data was also consistent with self-recognition accuracy for the action types (see supplementary materials S8 Text in S1 File for detail on confidence results).

Linear mixed modeling was implemented to analyze the effects of action type and viewpoint orientation on self-recognition performance. In addition to modeling the individual effects and their interaction term, we included random intercepts for both individual action variability and participant variability. The mixed model showed a significant main effect of action type, *F*(2, 24) = 11.42, p = < .001, but no effect of viewpoint orientation, *F*(1, 8048) = 1.24, *p* = 0.287, and no interaction between action type and viewpoint orientation, *F*(2, 8048) = 2.00 *p* = 0.09, *R*^2^_marginal_ = 1.90%, *R*^2^_conditional_ = 9.90%.

Bonferroni-corrected pairwise comparisons for the levels of action type revealed higher self-recognition accuracy for complex (*M* = 0.542, *SE* = 0.034) relative to simple actions (*M* = 0.406, *SE* = 0.034), *t*(24) = 4.02, *p* < .001) as well as relative to imitation actions (*M* = 0.398, *SE* = 0.034), *t*(24) = 4.25, *p* < .001. No difference was found between simple and imitation actions, *t*(24) = 0.237, *p* = 0.814, nor between the marginal means for viewpoint orientations i.e., facing left: 225° (*M* = 0.45, *SE* = 0.128), front: 0° (*M* = 0.45, *SE* = 0.128), right: 45° (*M* = 0.44, *SE* = 0.128), *p*s > .05. The finding is consistent with a previous study showing that self-recognition of walking actions is based on object-centered representations, independent of the viewing angle [16].

### Impact of individual actions on self-recognition performance

**Fig 5:**
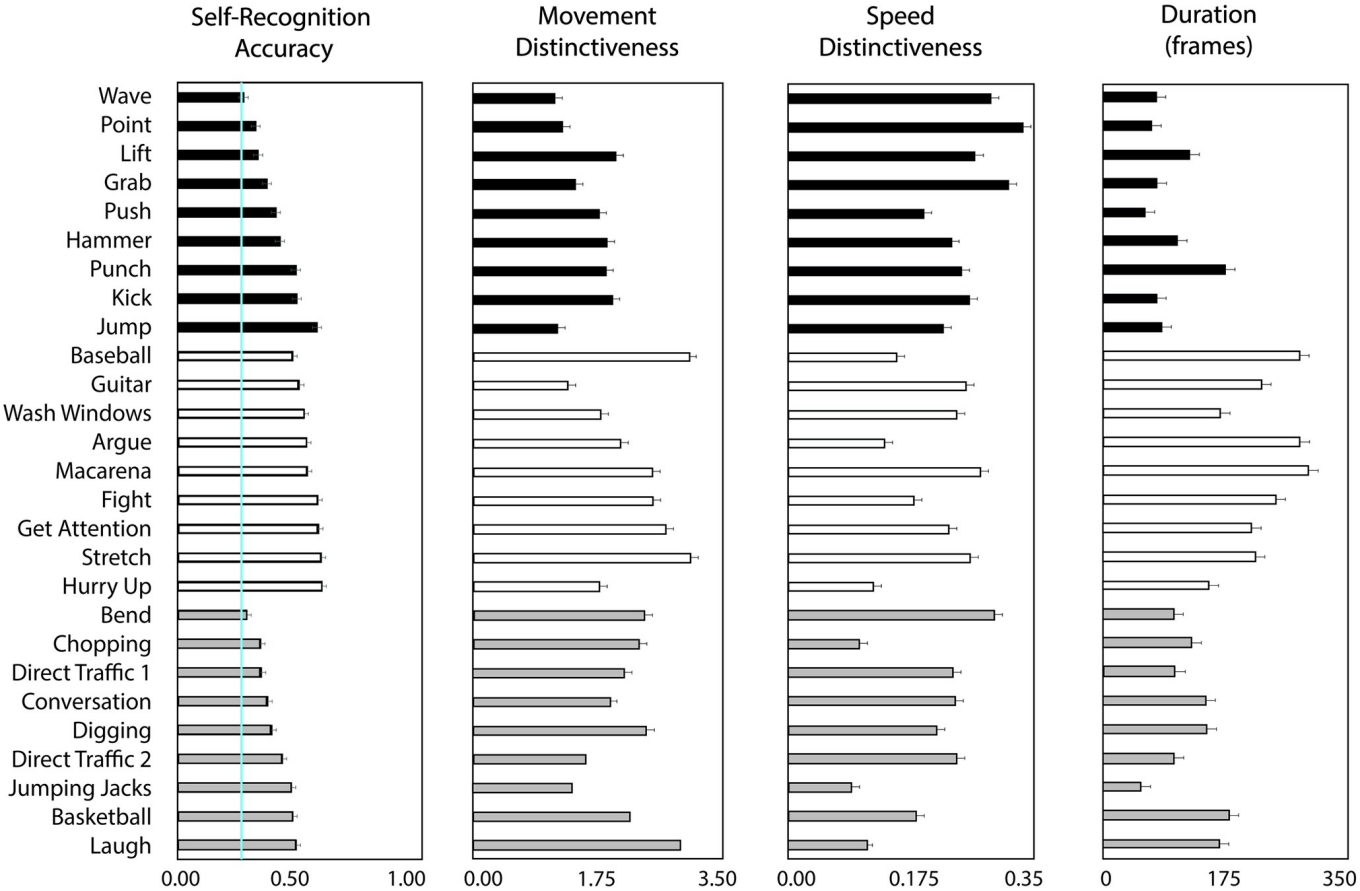
Results of the item-level analysis. *Left*: Results of self-recognition accuracy as a function of the individual action. Blue line indicates chance performance (0.25). *Middle Left*: Results of movement distinctiveness between individuals a function of the individual action. *Middle Right*: Results of speed differences between individuals as a function of the individual action. *Right*: Results of action duration differences between individuals as a function of the individual action. Color-coding of bars corresponds to action type: simple actions denoted in black, complex actions denoted in white, imitation actions denoted in gray. Error bars denote standard error of the means.

Analysis of item-level variability in self-recognition performance for individual actions (Fig 5) revealed that self-recognition performance varied in a large range of .27 to .59 across the 27 actions (with the chance level of .25 for recognizing self from four actions in the experiment). All complex actions (*stretch*, *get attention*, *wash windows*, *argue*, *guitar*, *hurry up*, *fight*, *baseball*, and *macarena*) were self-recognized significantly above chance (*ps* < .001). Most simple actions (*point*, *punch*, *lift*, *grab*, *push*, *hammer*, *jump*, and *kick*) except *wave*, and most imitation actions (*basketball*, *conversation*, *directing traffic p1*, *directing traffic p2*, *chopping*, *digging*, and *laugh*) except *bend*, were also self-recognized significantly above chance performance (*ps* < .030).

Based on the variability in self-recognition performance for individual actions, we examined the contribution of stimulus-level features of the actions to self-recognition. We derived difference indices between self-actions and actions performed by the distractor actions using the following features: action duration, speed of movements, and visual distinctiveness of movement trajectories. Specifically, we examined how self-action recognition performance was influenced by the action dissimilarity relative to the three distractor actors that performed each action during the self-recognition task. Greater feature distance values indicate higher dissimilarity, suggesting more distinctiveness of own actions. We used the above stimulus metrics (action duration, DTW-based movement distinctiveness, speed), and computed the minimum difference between an action performed by the participant and the three corresponding distractor actions for each trial. We modeled their contributions using linear mixed modeling. “Action type” (simple, complex, and imitation) and action difference indices of “action duration”, “speed”, “DTW trajectory” for each individual action were entered into the model as fixed factors, along with three two-way interactions between “action type” and each of the three difference features. The model also included “individual actions” and “participant” as random factors to allow variation in their intercepts. The model produced a significant effect of action type, *F*(2,31.82) = 14.033, *p* < .001, as well as a significant negative influence (*ß* = -0.025) of speed distinctiveness, F(1, 51.88) = 5.589, *p* = .021 on self-recognition performance. No significant effects were found for any other features (duration: *F*(1, 2692) = 0.53, *p* = .466; DTW: *F*(1, 468) = 0.450, *p* = .501) nor their interactions (speed x action type, *F*(2, 52.33) = 2.38, *p* = .103; DTW x action type, *F*(2, 553.95) = 1.64, *p* = .194, duration x action type, *F*(2, 2687.81) = 0.91, *p* = .401).

### Impact of motor experience to self-recognition performance

**Fig 6:**
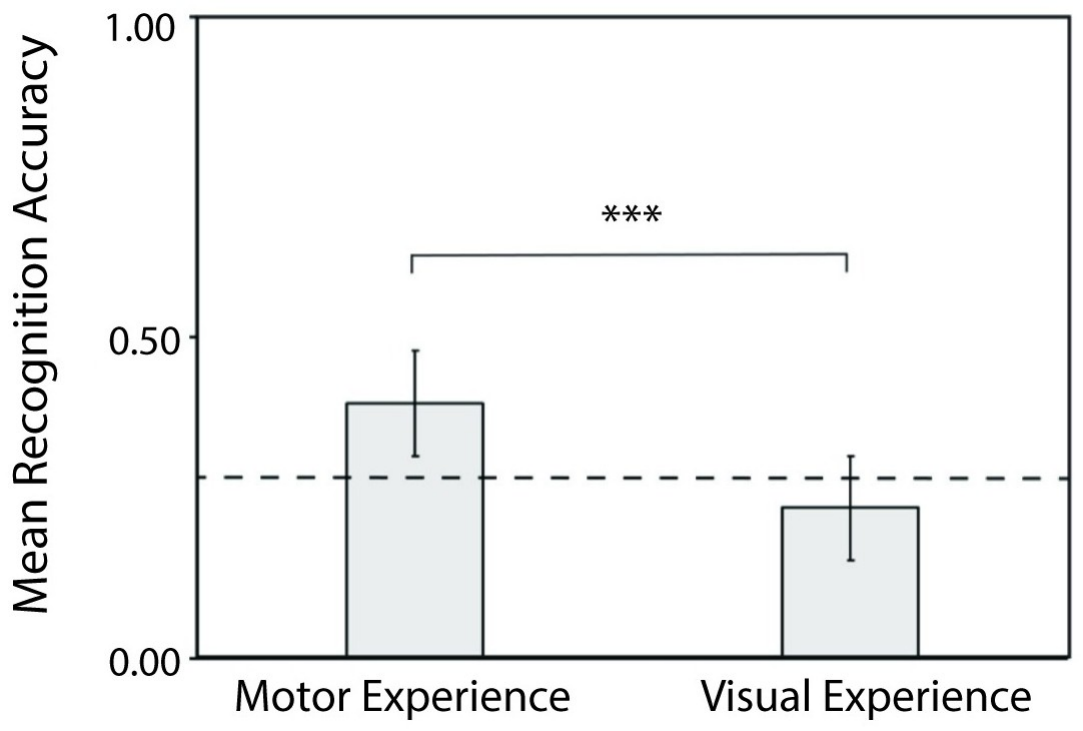
Recognition accuracy for imitation actions in the self-recognition task with motor experience versus performance in the visual-recognition task with visual experience. Significantly weaker performance from recognizing actions from visual experience than for self-recognition from performed actions. Dashed line indicates chance performance (0.25). Error bars indicate standard error of means.

Since we found sparse evidence in support of visual idiosyncrasies influencing self-recognition performance, but significant influences of action type, we sought to further measure the influence of motor experience on self-recognition performance. Thus, we contrasted performance in the self-recognition task relative to performance in the visual recognition task for imitation actions. Our task manipulation allowed us to compare between actions that participants previously performed (their own imitation action) versus actions that the participant observed during the motion recording session of another stick figure performing the imitation action. As shown in Fig 6, participants performed at chance level (0.25) for visual recognition of previously viewed actions (*M* = 0.23, *SD* = 0.19), *t*(72) = − 0.718, *p* = .475, *d* = .084, but showed much greater recognition performance in identification of one’s own action (*M* = 0.38, *SD* = 0.15), with visual recognition performance significantly lower than performance in self-identification, *t*(72) = 5.41, *p* < .001, *d* = .633. The around-chance performance for actions with only visual experience suggests that prior visual experience alone does not suffice for self-recognition. Instead, self-recognition may rely on action mechanisms that involve greater recruitment of the motor system [96].

### Impact of individual differences on self-recognition performance

The second analysis step focused on the impact of individual difference measures on self-action recognition. Two participants did not complete the VMIQ-2, and one participant did not complete the AQ questionnaire due to time constraints in the experimental session. Hence, listwise deletion resulted in 98 participants included in the individual differences analysis. Descriptive statistics for the scores of autistic traits (AQ score), schizotypal traits (SPQ score) and motor imagery ability (VMIQ-2 score), and subscale scores are reported in Table 1.

**Table 1:** Descriptive statistics for all composite (AQ, SPQ, and VMIQ-2) and subscale measures.

Measure	*N*	Max Score	Mean	Standard Deviation	Range
**AQ**	98	50	18.62	6.03	8–38
AQ-Comm	98	10	2.49	1.93	0–8
AQ-SocialSkill	98	10	2.49	2.21	0–10
AQ-Imagination	98	10	2.67	1.45	0–7
AQ-AttentionDetail	98	10	5.36	2.18	0–10
AQ-AttentionSwitch	98	10	5.60	1.95	1–9
**SPQ**	98	74	23.54	11.14	3–56
SPQ-UnusualPerceptual	98	9	2.56	1.95	0–9
SPQ-IdeasReference	98	9	3.81	2.39	0–9
SPQ-OddBeliefs	98	7	1.15	1.39	0–5
SPQ-Suspiciousness	98	8	2.66	1.96	0–8
SPQ-SocialAnxiety	98	8	4.09	2.33	0–8
SPQ-NoCloseFriends	98	9	2.29	2.20	0–9
SPQ-OddBehavior	98	7	1.15	1.99	0–7
SPQ-OddSpeech	98	9	3.39	1.93	0–9
SPQ-ConstrictedAffect	98	8	1.75	1.74	0–7
**VMIQ-2**	98	180	67.95	20.16	36–157
VMIQ-E	98	60	26.13	10.19	12–49
VMIQ-I	98	60	20.70	7.32	12–49
VMIQ-K	98	60	21.11	8.64	12–60

AQ (-Comm): Autism Quotient (Communication subtype), (-Detail): Attention to Detail subtype, (-Switching): Attention to Switching subtype; SPQ (-UnusualPerceptual): Schizotypal Personality Quotient (Unusual Perceptual Experiencse subtype), (-IdeasReference): Ideas of Reference subtype; VMIQ-2 (-E): Vividness of Motor Imagery Questionnaire (External subtype), (-I): Internal subtype, (-K): Kinesthetic subtype

First, regression analyses were conducted to examine relations between composite scores of individual difference measures and self-recognition performance. For each action type (simple, complex, and imitation), self-recognition performance was set as the dependent variable and three composite scores (AQ for autistic traits, SPQ scores for schizotypal traits, and VMIQ-2 scores for motor imagery traits) were included as predictor variables in the regression analysis. Nonparametric correlation analyses revealed significant relationships between some composite and subscale individual difference measures and self-recognition (reported in Table 2).

**Table 2:** Significant spearman rank-order correlations amongst task and selected trait measures.

Performance relationship between action type and trait measures	Spearman’s rho
Imitation Actions & VMIQ-2	ρ = -.221*
Imitation Actions & AQ-Comm	ρ = -.229**
Simple Actions & AQ-Comm	ρ = -.229*
Complex Actions & SPQ-Unusual	ρ = 0.264**

AQ (-Comm): Autistic Quotient (Communication subtype), SPQ (-Unusual): Schizotypal Personality Quotient (Unusual Perceptual Experiences subtype), VMIQ-2: Vividness of Movement Imagery Questionnaire

* *p* < 0.05

** *p* < 0.01

**Fig 7:**
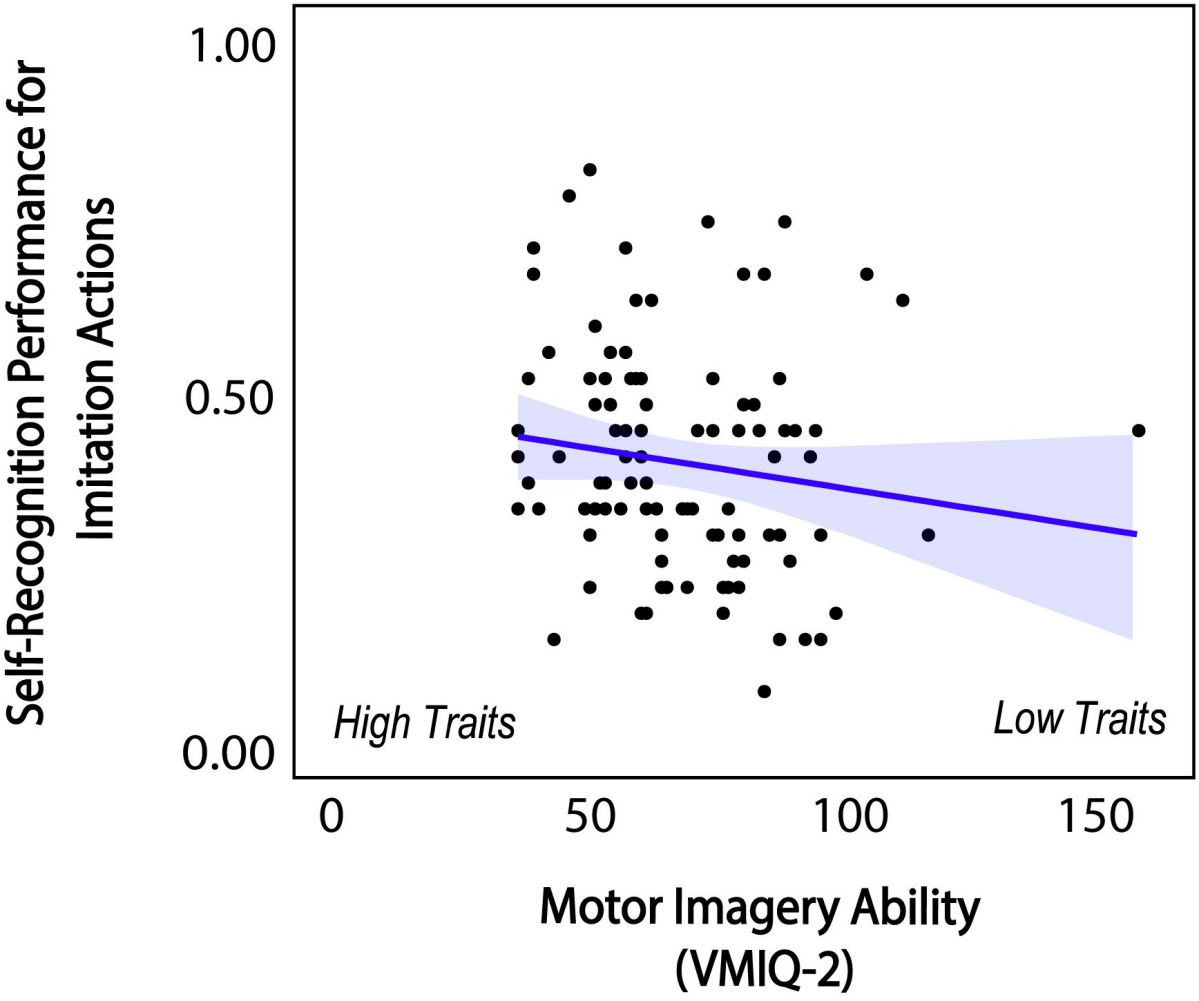
Significant relationship between composite motor imagery ability and self-recognition for imitation actions and 95% confidence interval. Blue line indicates regression slope. Note that lower VMIQ-2 score indicates better motor imagery ability.

For the individual difference measures, we found that composite motor imagery (VMIQ-2) scores negatively correlated with self-recognition performance for imitation actions (spearman *ρ* = -0.221, 95% CI [-.406, -.017], *p* = .029) (see Fig 7). Note that lower VMIQ-2 scores indicate stronger motor imagery ability. Thus, the negative correlation between motor imagery scores and self-recognition performance for imitation actions suggests that individuals with greater motor imagery ability were better able to recognize their own imitation actions. We did not find significant relationships between the other two composite measures (AQ and SPQ scores) and self-recognition performance.

Next, we examined whether subscale scores in the individual difference measures further related to self-recognition performance. We examined subscale scores for two main reasons. First, both ASC and SSC are characterized by multidimensional symptom expression, which may be masked by only examining the composite measure that averages across this variability. Second, selected subscale scores in both autistic and schizotypal traits are related to atypical biological motion processing [e.g., 68, 75] as well as disturbances in self-processing [60, 62, 97]. Considering the large number of possible predictors (5 subscales in AQ, 9 subscales in SPQ, and 3 subscales in motor imagery VMIQ-2), we used stepwise regression to select the important predictors in relation to self-recognition performance for the three action types (including all possible predictors in the model). Stepwise regression was used as the predictor-selection step since our individual difference analysis was exploratory. Additionally, the high collinearity between the measures (shown in Table 3; heatmap visualized in S1 Fig in S1 File), coupled with the small sample size, require a predictor selection step that can select the most informative variables, while also avoiding overfitting. After predictor selection, we computed spearman correlations as descriptive statistics to explore the relation between self-recognition performance and subscale individual differences.

**Table 3:** Spearman rank-order correlations amongst distinct composite and subscale measures.

Correlated Measures	Spearman’s Rho
**[AQ] & [SPQ]**	.502**
**[AQ]** & SPQ-Ref	.265**
**[AQ]** & SPQ-Anxiety	.352**
**[AQ**]& SPQ-OddBehav	.277**
**[AQ]** & SPQ-NoFriends	.451**
**[AQ]** & SPQ-OddSpeech	.281**
**[AQ]** & SPQ-Constrict	.477**
**[AQ]** & SPQ-Sus	.345**
**[SPQ]** & AQ-Attention	.351**
**[SPQ**] & AQ-Detail	.243*
**[SPQ]**& AQ-Comm	.313**
**[SPQ]** & AQ-Social	.493**
SPQ-Ref & AQ-Detail	.369**
SPQ-OddBehav & AQ-Social	.307**
SPQ-NoFriends & AQ-Social	.627**
SPQ-OddSpeech & AQ-Attention	.298**
SPQ-OddSpeech & AQ-Comm	.263**
SPQ-OddSpeech & AQ-Social	.215*
SPQ-Constrict & AQ-Attention	.301**
SPQ-Constric & AQ-Comm	.421**
SPQ-Constric & AQ-Social	.601**
SPQ-Sus & AQ-Attention	.309**
SPQ-Sus & AQ-Detail	.295**
**[VMIQ]** & AQ-Comm	.238*
VMIQ-E & AQ-Comm	.235 *

Autism-Spectrum Quotient [AQ]. AQ (-Comm): social communication subtype; AQ (-Detail): attention to detail subtype; AQ (-Social): social skills subtype; AQ (-Attention): attention switching subtype; Schizotypal Personality Quotient [SPQ]. SPQ (-Ref): ideas of reference subtype; SPQ (-OddSpeech): odd speech subtype; SPQ (-NoFriends): no friends subtype; SPQ (-Constrict): constricted affect subtype; SPQ (-Anxiety): social anxiety subtype; SPQ (-Sus): suspiciousness subtype; SPQ (-OddBehav): odd behavior subtype Vividness of Movement Imagery [VMIQ]. VMIQ (-E): external subtype. Composite measures are denoted in brackets and bolded.

* *p* < 0.05

** *p* < 0.01

**Fig 8:**
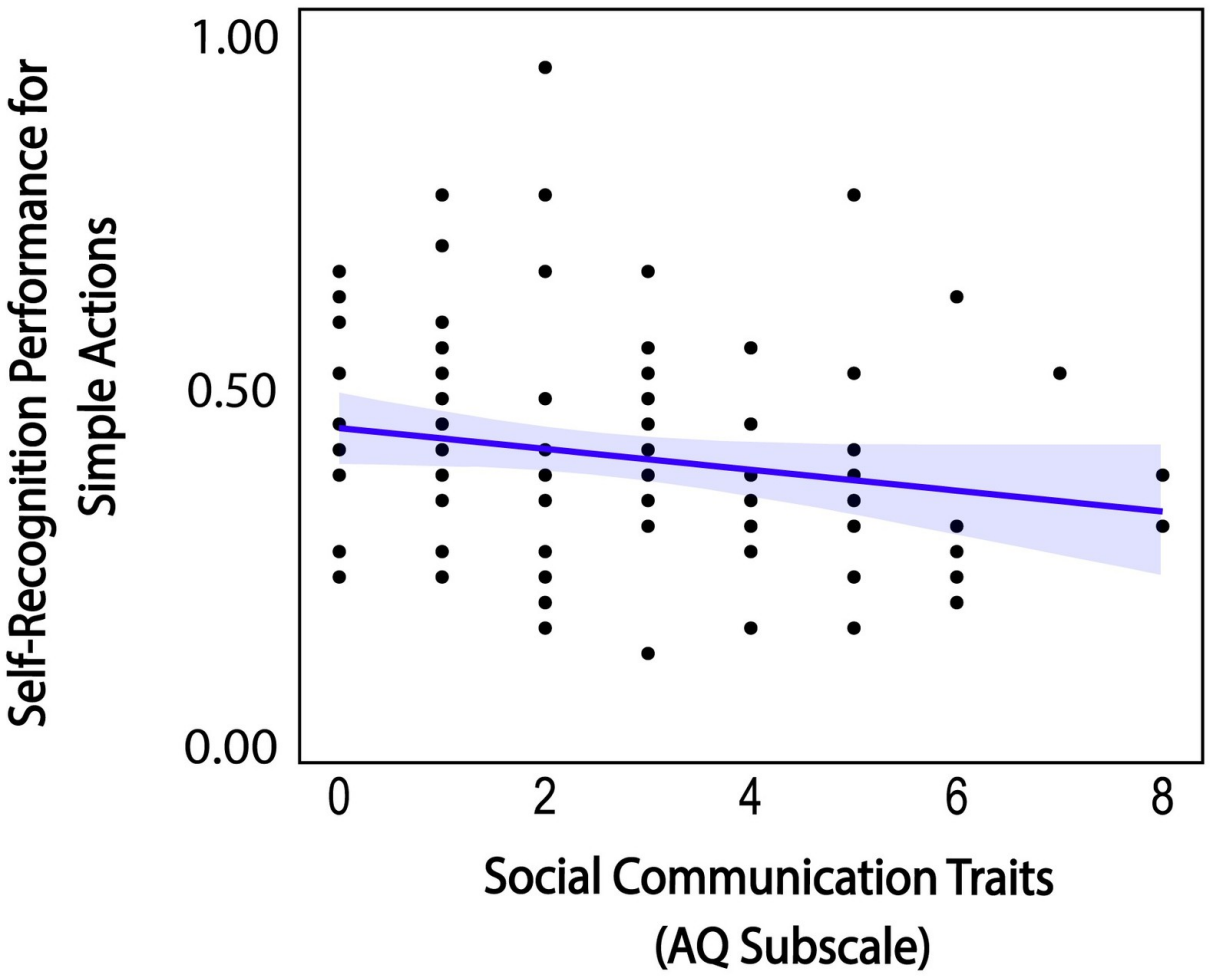
Relationship between AQ social communication subscale scores and self-recognition for simple actions and 95% confidence interval. Blue line indicates regression slope. Higher AQ scores indicate more autistic traits.

For the simple action condition, the stepwise regression selected a model with four subscale scores as strong predictor variables (AQ *social communication*, and three SPQ subscale scores including *odd behavior*, *ideas of reference*, and *no close friends*), (*F*(4,93) = 2.817, *p* = 0.030) adjusted *R*^2^ = .070, showing no multicollinearity (AQ *social communication*, *Tolerance* = .836, *VIF* = 1.196; SPQ *odd behavior*, *Tolerance* = .846, *VIF* = 1.182; SPQ *ideas of reference*, *Tolerance* = .842, *VIF* = 1.187; SPQ *no close friends*, *Tolerance* = .770, *VIF* = 1.299). However, among the four selected subscale scores, only AQ *social communication* scores revealed a significant coefficient (*t*(92) = -2.672, *p* = .009). Nonparametric correlation analysis confirmed the negative relation between AQ *social communication* and self-recognition for simple actions (spearman *ρ* = -.229, 95% CI [-0.414, -0.026], *p* = .023) (Fig 8). As higher AQ scores indicate more autistic traits [83], the negative correlation indicates that participants with more autistic traits in *social communication* were less able to recognize their actions when the actions conveyed simple types of goals with less motor planning.

**Fig 9:**
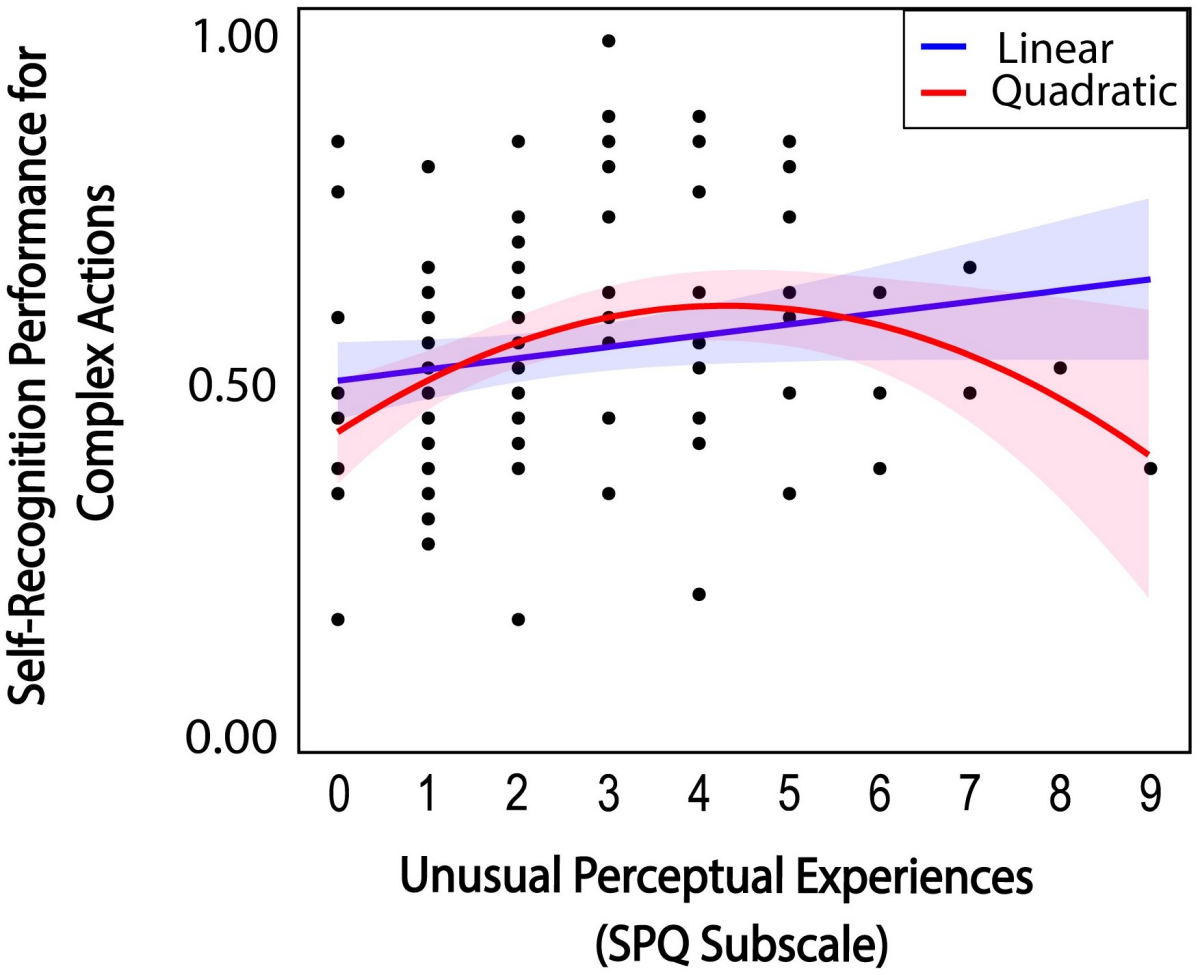
Relations between SPQ unusual perceptual experience subscale scores and self-recognition for complex actions and respective 95% confidence intervals. Higher SPQ scores indicate more schizotypal traits.

For complex actions involving more motor planning (S5 Table in S1 File), the stepwise regression selected a model with three SPQ subscale scores as predictor variables (including *unusual perceptual experiences*, *odd behavior*, *ideas of reference* and *no close friends)* in relation to self-recognition performance. The selected model did not reach statistical significance (*F*(3,94) = 2.283, *p* = 0.084). On further examination, we noticed a quadratic trend between SPQ *unusual perceptual experience* scores and self-recognition for complex actions. Thus, we introduced an additional predictor variable of quadratic SPQ subscale scores of *unusual perceptual experiences* to the stepwise regression analysis. The results converged to a significant model with two predictor variables (SPQ *unusual perceptual experience* subscale scores and its quadratic term, *Tolerance* = .110, *VIF* = 9.054), *F*(2,95) = 5.782, *p* = 0.004, adjusted *R*^2^ = .056. As shown in Fig 9, we observed a significant relationship between the SPQ *unusual perceptual experiences* subscale and self-recognition performance for complex actions (spearman *ρ* = 0.264, 95% CI [.064, .445], *p* = .008). In addition to the linear relation, self-recognition performance for complex actions also related to a quadratic trend of SPQ *unusual perceptual* scores, revealed by significant coefficients to the quadratic term (*t*(92) = -2.937, *p* = .004). The coexistence of linear and quadratic relations indicates that participants with mid-range SPQ *unusual perceptual experiences* scores showed best self-recognition performance for complex actions.

For imitation actions (S6 Table in S1 File), the stepwise regression analysis selected a model with six predictor variables, including AQ *social communication (Tolerance* = .756, *VIF* = 1.322*)* and *attention switching (Tolerance* = .797, *VIF* = 1.255) subscale scores, three SPQ scores of *odd speech (Tolerance* = .747, *VIF* = 1.338), *suspiciousness (Tolerance* = .692, *VIF* = 1.446), and *no close friends (Tolerance* = .737, *VIF* = 1.357), and VMIQ-2 *Kinesthetic* scores *(Tolerance* = .976, *VIF* = 1.024), in relation to self-recognition performance (*F*(6,91) = 2.895, *p* = 0.013), adjusted *R*^2^ = .105. However, only AQ *social communication* and *SPQ suspiciousness* scores showed significant coefficients (*t*(90) = -3.286, *p* = .001; *t*(90) = − 2.336, *p* = .022). The confirmatory nonparametric correlation analysis revealed a significant relation between self-recognition performance for imitation actions and the AQ *social communication* subscale scores (spearman *ρ* = -0.229, 95% CI [–0.414, -0.026], *p* = .023). This result indicates that people with higher AQ traits associated with social communication ability performed worse in the self-recognition task for imitation actions.

As self-recognition performance for imitation actions was also correlated with the VMIQ-2 composite scores of motor imagery ability reported earlier, we ran a second stepwise regression analysis with four predictor variables, including AQ *social communication* scores and all three VMIQ-2 subscale scores. This stepwise regression selected a model with two predictor variables, AQ *social communication* scores *(Tolerance* = 1.00, *VIF* = 1.00) and VMIQ-2 *kinesthetic* motor imagery scores *(Tolerance* = 1.00, *VIF* = 1.00), *F*(2,95) = 3.652, *p* = .030, adjusted *R*^2^ = .052.

**Fig 10:**
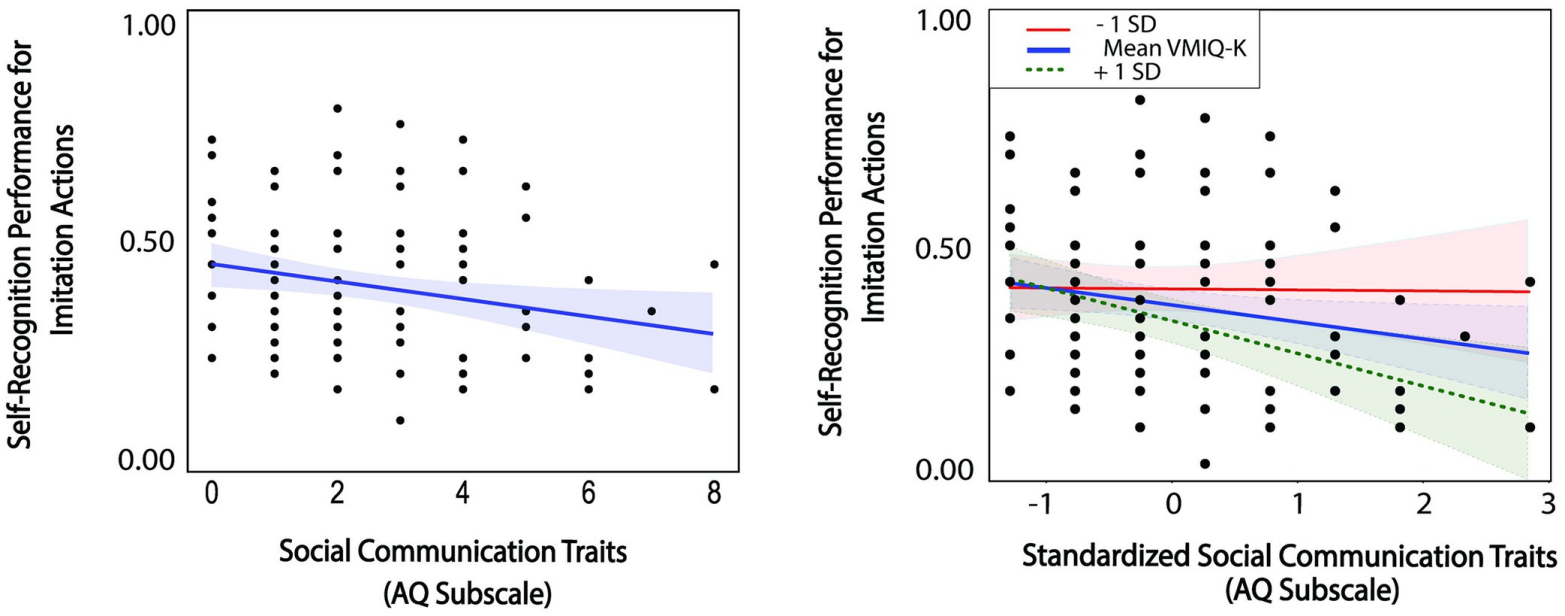
Relations between self-recognition for imitation actions with AQ *social communication* subscale scores (left panel) and moderation of this relationship by kinesthetic motor imagery ability (right panel). Right panel: Individual slopes were significant for those with low kinesthetic motor imagery ability (+ 1 *SD*) and mean imagery ability, but not high ability (- 1 *SD*). Note that higher AQ scores indicate more autistic traits, and higher VMIQ-K scores indicate weaker imagery ability.

Based on the joint influence of AQ *social communication* and VMIQ-2 on self-recognition for imitation actions, we examined associations between these individual differences and imitation actions. As shown in the right panel of Fig 10, the negative relationship between AQ *social communication* scores with self-recognition performance for imitation actions was moderated by kinesthetic motor imagery ability (*ß* = − 0.032, 95% CI [-0.063, -0.001], *p* = .043): higher *kinesthetic* motor imagery ability (- 1 *SD*: *VMIQ scale reversed*) did not influence self-recognition (*p* = .949). However, having average (*ß* = -0.034, 95% CI [-0.065, -0.027], *p* = .034) or lower *kinesthetic* motor imagery ability (+ 1 *SD*) (*ß* = 0.066, 95% CI [-0.108, -0.024], *p* = .003) significantly reduced self-recognition performance as AQ *social communication* traits increased. This result suggests that *kinesthetic* motor imagery ability may be an important compensatory marker in the autism spectrum for supporting self-recognition from actions. A full table of all regression results are reported in supplementary materials: S4–S7 Tables in S1 File.

## Discussion

On a large sample of participants, we measured the underlying factors that contribute to performance variability in self-action recognition. First, we found that self-recognition was primarily influenced by the type of action performed, linked to the complexity of motor planning required during action production. Next, we used quantitative measures of action similarity, which showed that visual distinctiveness of movements did not primarily influence self-recognition performance. Finally, we found that individual differences in motor imagery ability and in subscales of autism-spectrum and schizotypy influenced self-recognition performance for distinct action types. Specifically, composite motor imagery traits and subscale traits linked to kinesthetic motor imagery, autism-spectrum (AQ social communication) and schizotypy (SPQ unusual perceptual experiences) were associated with self-recognition for different action types.

### The degree of motor planning influences self-recognition performance

Our first finding replicated prior research that participants self-recognize actions based on the kinematics in point-light displays [1, 2, 17, 18]. We extended the literature to show self-recognition performance also varied according to the degree of motor planning, delineated by our motoric factor of action type (simple, complex, and imitation) (similar to [17]). A few prior studies have focused on item-level delineations based on perceptual variability or movement idiosyncrasies (e.g., [2]). However, the perceptual consequences of self-generated actions are driven by motor planning during action performance [8], and hence causally vary as a function of motor planning during action production (e.g., [31]). We characterized complex actions as having the greatest degree of motor planning due to the more complex variability of the goal representation space (relative to simple actions) (e.g., [31]), and imitation actions having the least motor planning due to mimicking another person’s movement patterns [73, 98].

Across individuals, we found consistent evidence that self-recognition performance varied according to action type. Specifically, the self-recognition performance pattern mostly mapped onto the motor planning differences, with greatest self-recognition performance for complex actions (most motor planning) relative to simple or imitation actions. No significant differences in self-recognition accuracy were observed between simple and imitation actions, which we attribute to the variability in motor complexity for actions within the imitation action category. This may have reduced the impact of motor planning differences between simple and imitation actions. Notably, the performance pattern did not relate to trajectory differences observed for movement idiosyncrasies computed by DTW, nor to speed or duration differences. Although we cannot completely rule out the impact of some other stimulus-level features on self-action recognition, the degree of motor planning involved during action production does provide a parsimonious account for the variability in self-recognition performance observed in the current study.

### Visual distinctiveness does not primarily contribute to self-recognition performance

What mechanisms could influence the performance variability for self-recognition of the action types? Prior work has speculated on two main contributors to self-recognition of actions. First, proprioceptive and kinesthetic information/memory retained from executing familiar motor actions (i.e., motor experience) during action performance may play a strong role in influencing self-action recognition. During action recognition, this may be understood as the comparison between an offline, kinesthetic memory-based action representation (or action schema) [99] to the online perception of the point-light actions. This mechanism may be recruited to facilitate a matching process to recognize an action as one’s own. On the other hand, participants may recognize their movements in a predominantly visual manner during action recognition, which need not rely on comparisons with stored action schemas, but rather on visually identifying idiosyncratic movement features, such as perceptual distinctiveness (e.g., speed of movements; motion trajectories in observed actions) that may “pop out” when viewing the stimulus. Our study sought to measure the individual contributions of these accounts to self-action recognition.

We used quantitative measures of action similarity to first measure the contribution of visual experience to self-recognition. We computed visual distinctiveness for three different indices related to speed, action duration, and movement similarity. Across the three indices, only a moderate effect of speed distinctiveness was found on self-recognition performance. However, this effect showed a negative trend, suggesting that more distinct speeds of actions actually impaired self-recognition performance. Moreover, this effect did not interact with the action type factor, which provides an unlikely explanation for how action type influences self-recognition performance. No other relationships were found between self-recognition for the action types and differences in movement similarity using DTW nor action duration. Based on these indices, our results suggest that visual distinctiveness is unlikely to primarily contribute to self-action recognition, when accounting for the action type.

To assess the contribution of motor experience to self-recognition, we also included a visual recognition task for imitation actions. Here, participants had to recognize the actor’s movements that they had previously seen and imitated. Performance on this task showed chance-level recognition of briefly observed visual actions that participants had previous visual experience with, as compared to significantly above-chance self-recognition performance for actions that participants had previous motor experience with. Moreover, all actions, regardless of their degree of complexity, showed viewpoint-invariant self-recognition (frontal and right/left half-profile) of identifying own actions, confirming a body-centered representation for self-generated actions [16]. Together, these results underscore the contribution of motoric factors to visual self-recognition.

### Individual differences influence self-recognition performance for distinct action types

Beyond the influence of action characteristics on self-recognition, our analyses also revealed associations between the action type and individual differences linked to motor imagery ability and subclinical subscales (AQ social communication, and SPQ unusual perceptual experiences). For imitation actions, composite motor imagery scores significantly correlated with self-recognition performance. Motor simulation theories [35, 96] posit the central role of motor imagery during action perception. Our results suggest that a compensatory increase in motor imagery ability may be required to self-recognize one’s own imitation action, since these actions had the least amount of motor planning. That is, no explicit action label was provided during the action execution session; hence participants just copied the movements they observed.

No significant relationships were found between the visual imagery subtypes (internal and external) and self-recognition for any action types. This behavioral dissociation between visual and kinesthetic imagery converges with neural evidence revealing partially distinct brain regions for visual imagery (parieto-occipital) and kinesthetic imagery (i.e., parietal and premotor) [100]. It is possible that during the perception of one’s own action, previous motor experience may aid action simulation by using neural resources linked to kinesthetic motor imagery [101, 102] to simulate own actions.

We found a negative relation between the AQ social communication subscale and self-recognition performance for simple actions. This negative relationship was moderated by kinesthetic motor imagery traits: greater AQ social communication traits attenuated self-recognition performance for imitation actions, only when kinesthetic motor imagery ability was weakened (low or average). In other words, greater kinesthetic motor imagery ability could play a compensatory role in the Autism-Spectrum, evidenced by the reduced negative effects of AQ social communication on self-action recognition in individuals with more kinesthetic motor imagery traits. As motor imagery is thought to share neural resources with biological motion perception (e.g., [36]), one possibility for the negative AQ social communication relation could be due to difficulties in biological motion processing. This is consistent with neuroimaging evidence showing reduced brain activity in prefrontal and left temporal cortices in individuals with high AQ social communication traits when they viewed point-light actions [68]. Behaviorally, this interpretation also converges with empirical work linking the AQ social communication subscale to atypical processing of local kinematic information (e.g., individual joint motion) in biological motion [71]. Previous research in biological motion perception has shown that action recognition depends on both local processing of joint movements [103, 104] and top-down influences [105]. For simple actions with less perceptual variability, superior local processing of joint movements may be necessary to discriminate fine details of self from other actions, possibly compromised in people with high AQ social communication traits (corresponding to weakened social communication skills). By consequence, the nonsignificant relationship between AQ social communication traits and self-recognition performance for complex actions may result from richer action cues provided by complex actions given their relatively longer durations. Specifically, when there is more action information in the display, self-recognition should naturally be facilitated, regardless of the AQ spectrum. Note that the interpretation of weakened local processing in people with high AQ social communication traits contradicts a few behavioral patterns observed in biological motion research that have utilized the composite AQ measure. Many of these previous studies suggest the reverse pattern—superior local processing and a generalized disturbance in global processing for individuals with high degree of autistic traits. However, these findings remain inconsistent (largely attributed to task-specific and individual variability) (see detailed discussions in [70]). Our study introduces a possible reconciliation by exploring the subscales, rather than averaging across the spectrum.

Scores on SPQ unusual perceptual experiences were quadratically related to complex actions, such that low and high individuals performed worse at self-recognizing complex action sequences. How might the SPQ subscale modulate self-recognition for complex actions? Prior work suggests impaired action perception across the schizophrenia spectrum [102, 106]. However, we only observed a significant relationship with self-recognition performance on one action type (i.e., complex actions). Thus, the present results cannot be reduced to a generalized abnormality in biological motion perception. Rather, atypical action monitoring in suppressing the sensory consequences of action may produce false positives—misattributions that the action is externally generated [107]. The unusual perceptual subscale focuses on the positive (i.e., first-rank symptoms) of SSC, including incoherent perceptual experiences that reduce one’s sense of agency [65], blurring the boundaries between the self and other (e.g., [48, 108, 109]). In relation to atypical action monitoring, prior studies have shown that individuals high in SPQ unusual perceptual traits are more successful at self-induced tickling [67], likened to a disrupted match between predicted efference copies and sensorimotor outcomes. The action monitoring atypicality may be particularly exacerbated by complex actions that require a greater degree of motor planning and preparation. Weakened self-recognition performance on complex actions with those low on SPQ unusual perceptual experiences traits is less clear. It is possible that the weakened self-recognition may be due to decreased perceptual acuity in discerning subtle movement patterns, induced by the distraction of perceptual variance in complex action sequences. This could manifest in behavioral differences based on false negatives (i.e., decreased discrimination of perceptual idiosyncrasies), rather than on false positives (i.e., misattributions). Additionally, prior research has also shown an advantage in individuals with high schizotypal traits for tasks that involve visuospatial transformations. [109] found more accurate self-other perspective taking in individuals with high positive schizotypal traits on tasks that require adopting another person’s bodily perspective. It is possible that having reduced positive schizotypal traits could negatively affect this ability to recognize oneself in the third person, due to difficulties adopting another visuospatial perspective during identity recognition.

Neither composite subclinical measure (schizotypal or autistic) showed a relation to self-recognition performance. If our analysis was restricted to the composite measures, this would suggest that physical aspects of the self (i.e., bodily recognition) are intact in both conditions, consistent with many studies on visual self-recognition (e.g., [110–114]; cf. [72]). However, the clear pattern of relations with the subscales suggests that a focus on the composite measure could be misleading, as it masks psychopathological variance in the general population. SSC and ASC have heterogenous symptomatology, residing on a continuum that extends well into the general population, and frequently updated in light of DSM revisions. While models of AQ and SPQ symptomatology posit the large phenotypic overlap between AQ and SPQ subscales (which our results maintain), the AQ social communication and SPQ unusual perceptual experiences dimensions appear to reside on a diametric axis, largely exempt from the overlap [90, 115]. The present results confirm the diametric relation presented in the prior work, as no relationship was observed between the subdimensions of interest (i.e., AQ social communication and SPQ unusual perceptual experiences), which instead shared unique variance with each respective clinical composite measure. Since the composite measures average across the spectrum, a foundational approach for future individual difference studies should prioritize subscales, which may reveal finer-grained individual variability.

Our study presents some outstanding limitations. Though we separated actions based on motor planning, our actions included a range of social and non-social actions. In doing so, we preserved the naturalistic nature of the actions; however, additional studies can augment the work with more refined methods of controlling the action types. Another limitation pertained to the action similarity analysis in the present paper. The DTW analysis only derives similarity by comparing joint movements, not considering other factors that can influence similarity judgments such as body structure similarity across individuals, or semantic similarity across actions. In addition, DTW gives the same weight to each frame in the video. In some actions, subtle movement differences in a short period may play a more important role in determining action similarity than movements in other periods. These subtle movement segments, not capturable with dynamic time warping analysis across groups, may also be needed to assess whether motor performance differences are associated with our psychometric traits (e.g., [116, 117]) or may provide idiosyncratic cues to identity in other ways. Importantly, our results do not rule out that perceptual distinctiveness could indeed be a relevant cue to self-recognition, as was found in [29]. Rather, we consider perceptual variance to be a consequence of the underlying motoric complexity afforded by different action goals, which may be more negligible when the action type is accounted for. As [29] focused on simple postural movements, the goal complexity remained consistent across action sequences, which may explain why visual distinctiveness played a bigger role in self-recognition in their study. Additionally, we selected distractor actions that were matched to the participant’s biological sex. We acknowledge that this approach may not fully encompass the spectrum of gender, cultural, and sexual identity differences that could and should ideally be measured in relation to participant action variability. Future research could benefit from a more inclusive approach by including these demographic factors. Finally, our study is exploratory in examining the relations between self-recognition and individual differences. The correlation results were largely descriptive statistics and did not maintain significance after controlling for multiple comparisons using the Bonferroni correction (although the Bonferroni correction is excessively conservative). Future studies using grouped approaches (e.g., grouping participants with high and low degree of traits) may increase the power to detect the effects we found.

## Conclusion

Certain species are capable of self-recognition, while few develop motor-based, self-other mapping mechanisms in the brain. Visual self-recognition of point-light actions provides a unique lens to gauge the core perceptual and motor mechanisms underlying self-representations in humans. These paradigms control the level of visual familiarity people have with self-related stimuli (e.g., compared to familiar faces, body images, voices) from a third-person perspective, highlighting the importance of “acting” in “seeing” the self. Our findings complement a large body of work in self-processing, demonstrating that self-recognition is possible for stimuli even with little visual experience, influenced by our own motor experience.

## Supporting information

S1 File (DOCX)
