# Supplementary material for: Individual differences and motor planning influence self-recognition of actions
Source: PLoS One. 2024 Jul 30;19(7):e0303820. doi: 10.1371/journal.pone.0303820 (PMC11288417; doi:10.1371/journal.pone.0303820)
Supplement: S1 File — (DOCX) [file pone.0303820.s001.docx]

**Individual differences and motor planning influence self-recognition of actions**

# SI. INTRINSIC MEASURES

Independent sample t-tests revealed no significant effects of gender on composite AQ (*t*(96) = 1.06, *p* = .293, *d* = .239), SPQ (*t*(96) = 0.01, *p =* .994, *d* = .002), or VMIQ-2 (*t*(96) = -0.98, *p* = .328, *d* = -.222) scores. Figure S1 shows correlation heatmap between all intrinsic measures.


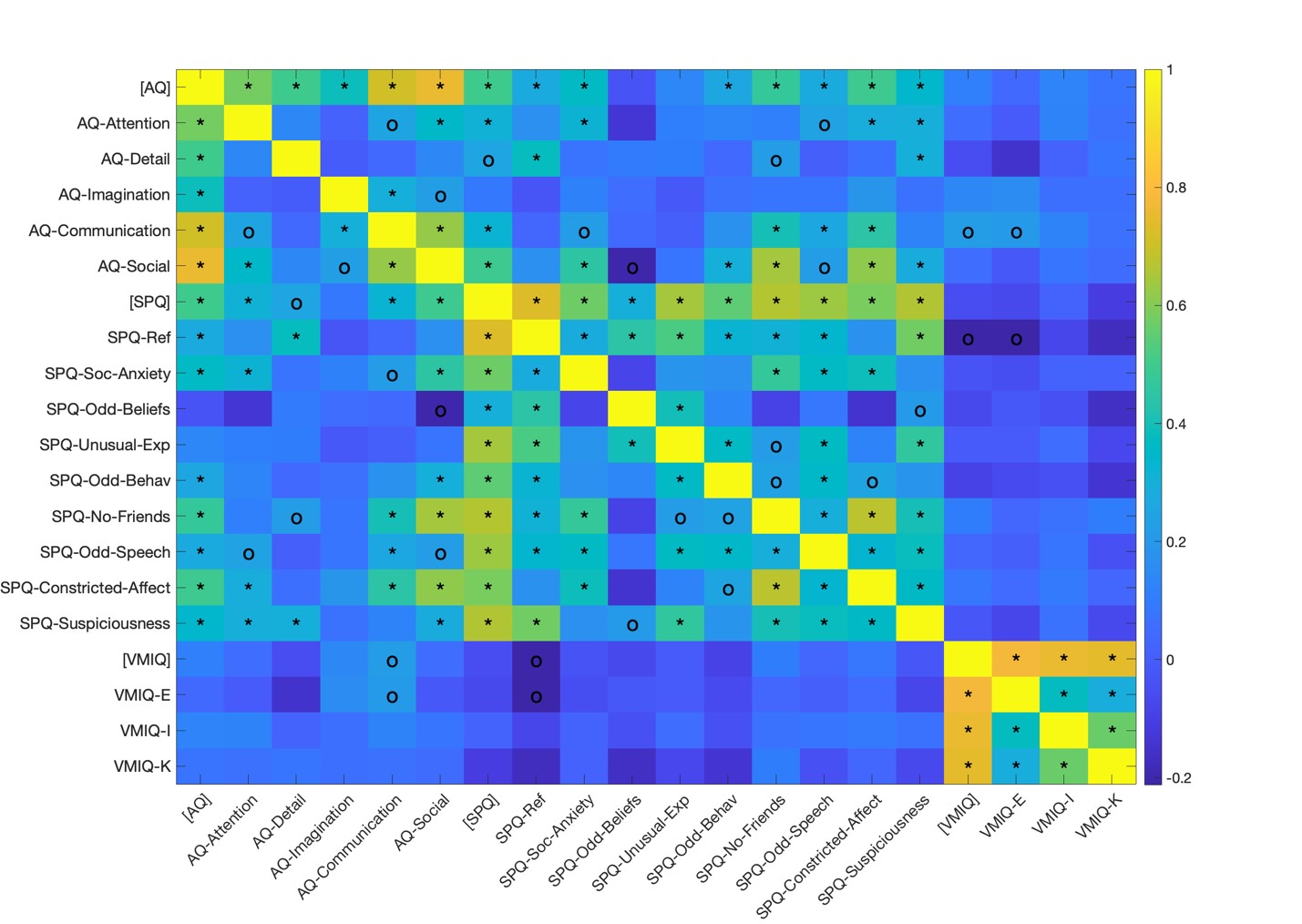


**Fig S1.** Spearman correlation heatmap depicts spearman rank-order relationships between all intrinsic measures (composite scores in brackets). Brighter colors indicate stronger relationships between trait measures. Circles denote .01< *p* < .05; asterisks denote *p* < .01 significance.

# SII. ANOVA VIEWPOINT RESULTS

To assess the influence of action viewpoint orientation on self-recognition, we computed both linear mixed modeling (reported in the manuscript) and an ANOVA (reported here). A two-way repeated-measures ANOVA with action types and viewpoint orientations as within-subjects factors on self-recognition performance revealed no effect of viewpoint orientation, facing left:

225° (*M* = 0.45, *SD* = 0.16), front: 0° (*M* = 0.45, *SD* = 0.14), right: 45° (*M* = 0.44, *SD* = 0.15), *F*(2, 200) = 1.58, *p*  = .207, η_p_^2^= .016. The finding is consistent with a previous study showing that self-recognition of walking actions is based on object-centered representations, independent of the viewing angle [1]. The analysis yielded a significant main effect of action type (simple, complex, and imitation), *F*(2, 200) = 43.09, *p* < .001, η_p_^2^ = .301. Bonferroni-corrected pairwise comparisons revealed that self-recognition was more accurate for complex than simple actions (*t*(100) = 7.86, *p* < .001, *d* = .782) and imitation actions (*t*(100) = 8.56, *p* < .001, *d* = .851). No difference emerged between simple and imitation actions (*t*(100) = 0.54, *p* = .593, *d* = .053). We also found a significant two-way interaction between orientation and action type (Figure 4 in main text), *F*(4, 400) = 2.77, *p* = .027, η_p_^2^ = .027, mainly driven by the weaker performance in the frontal view than side views for the imitation actions (*F*(2, 200) = 4.124, p = .018, η_p_^2^ = .040). However, the small effect size calls for caution in interpretation of this interaction effect, as the degree of visual ambiguity due to body occlusion likely impacted self-recognition performance for certain action orientations. Among the imitation actions, three actions (e.g., *bend, digging,* and *chopping wood*) consisted of the most self-occlusion from the frontal view, resulting in grouping ambiguity in the point-light display.

# SIII. LINEAR MIXED MODEL RESULTS

To measure the influence of stimulus-level features on self-recognition, we derived difference indices between self-actions and actions performed by other participants using the following features: action duration, speed of movements, and visual distinctiveness of movement trajectories of the performed actions. Greater feature distance values indicate higher dissimilarity, suggesting more distinctiveness of own actions. Across all features, nonparametric correlations revealed no significant relationships with self-recognition accuracy for the 27 actions (*duration:* spearman *ρ* = .012, *p* = .956; *speed*: spearman *ρ* = .082, *p* = .682; *movement distinctiveness*: spearman *ρ* = 0.053, *p* = .789).

Since actions performed by the participant relative to other participants may be too coarse a metric since participants never observed their own action against other participants’ actions in the task, we compared self-recognition performance against the three distractor actions that were shown in the experiment. In the manuscript, we report the distance between the participant relative to the minimum distance between distractors (i.e., comparing self-generated actions relative to the most similar distractor action shown on each trial). We chose to compare minimum distance rather than an average distance measure across all distractors, since self-recognition could be driven by the visual similarity between the participants’ actions and the most visually similar distractor action. Averaging across all three distractors could be uninformative as participants never viewed an averaged distractor action in the main task. Hence, we reported the minimum distance in the main text of the manuscript, and report here supplemental distinctiveness calculations based the average distance between the participants’ actions and the three distractor actions. Linear mixed modeling was implemented using the lme4 [2] and lmerTest [3] packages in R and significance of fixed effects was estimated using Satterthwaite’s approximation for degrees of freedom of F statistics. Planned post-hoc comparisons for the mixed model used the function emmeans() in R [4] with degrees of freedom adjusted using Kenward-Roger approximation. Models were estimated using restricted maximum likelihood.

Using average distance between the participant and all three distractors, we replicated our minimum distance findings, with a significant main effect of action type on self-recognition accuracy (action type, *F*(2,23.96) = 4.845, *p* = .017) and action speed differences *F*(1, 34.11) = 6.395, *p* = .016), which was weakly negative (b = -.0385). We observed a significant interaction between DTW movement differences and action type*, F*(2, 322.87) = 4.99, p = .007. As shown in Figure *S2,* self-recognition performance for simple actions was greater when the actions had more movement distinctiveness relative to the distractors (i.e., greater DTW difference) (r = 0.11, *p* = .001), but not for complex or imitation actions (*p*s > .05). Thus, movement distinctiveness appeared to help differentiate identity for simple actions, but not for the other action types. The influence of DTW distinctiveness for simple actions, suggests that movement distinctiveness improved self-recognition ability only for simple actions. Since simple actions are relatively hard to discern between individuals (simple actions goals with low DTW variability), visual distinctiveness may play a stronger role in aiding self-recognition for simple actions when only sparse visual information is available. No relation was observed between movement distinctiveness and either of the other two action types (complex or imitation). Together, the results confirm the robustness of action type as a primary influence on self-recognition performance.


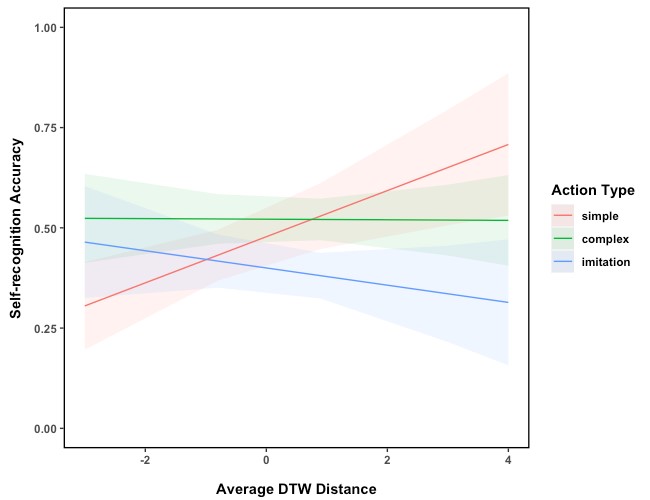


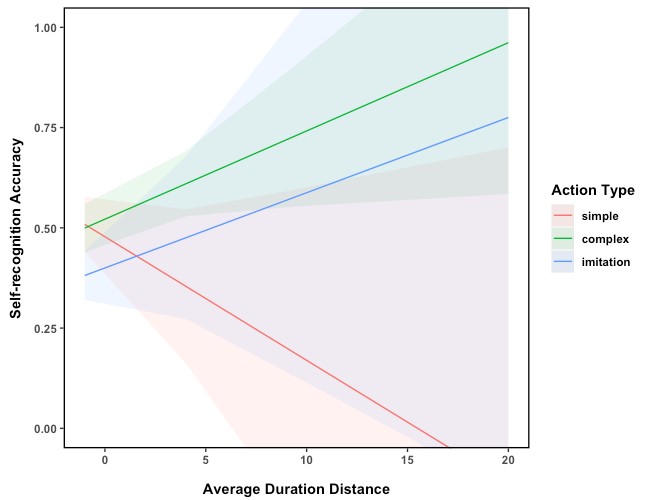

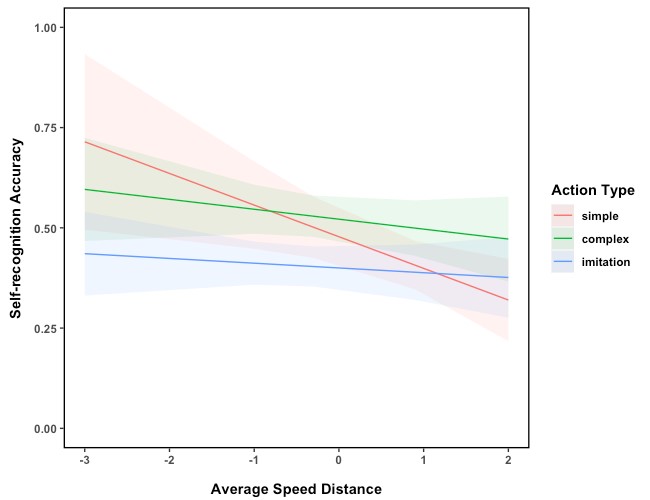


**Fig S3.** *Top Panel:* Distinctive movements measured by trajectory differences (DTW difference index) showed a positive relationship with self-recognition accuracy for simple actions (r = 0.11, p = .001), but not for complex and imitation actions (ps > .05). *Bottom:* Non-significant plots for duration distinctiveness (*left panel*) and speed distinctiveness (*right panel*).

# SV. REGRESSION TABLES

**Table S4.**


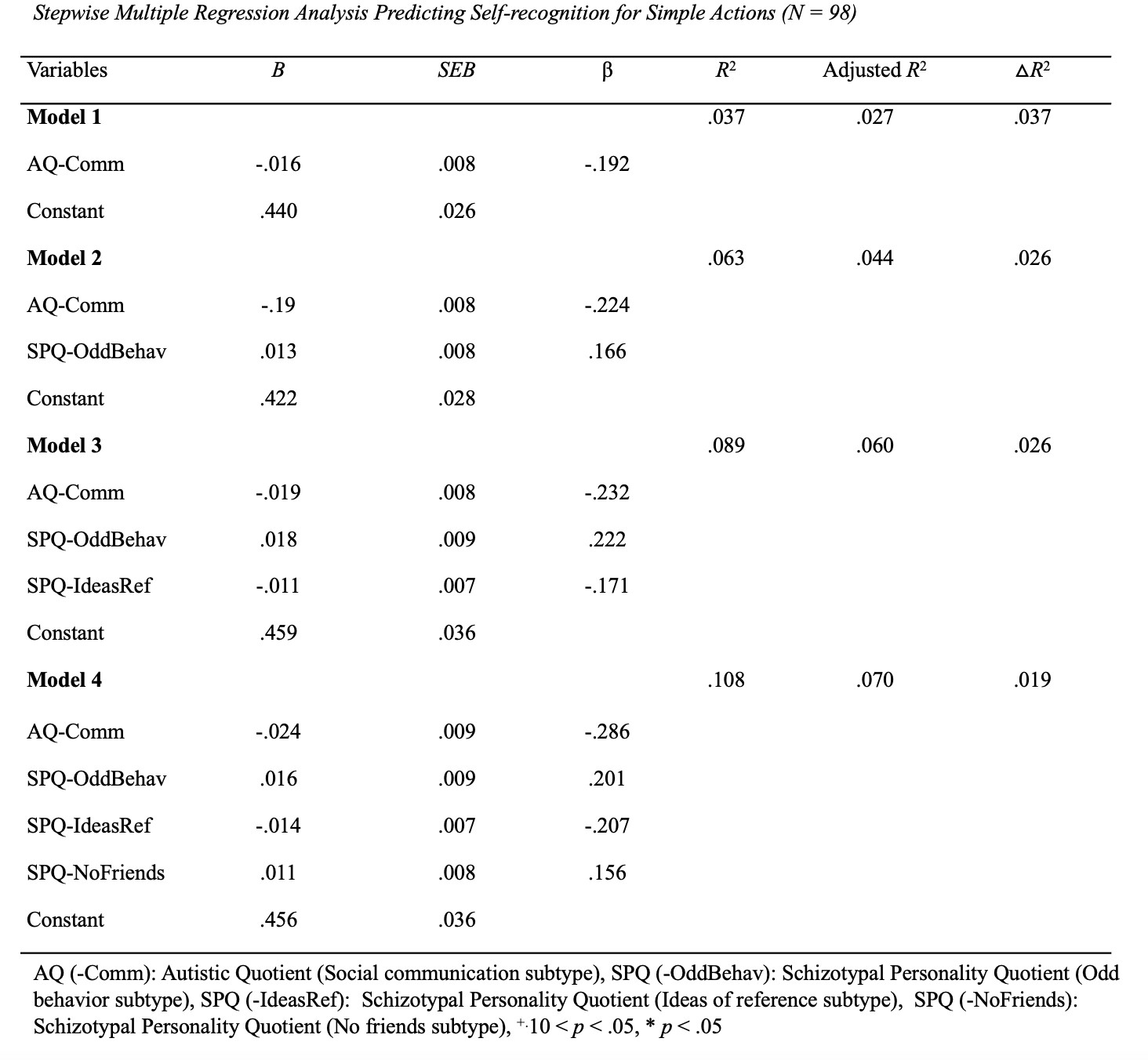


**Table S5.**


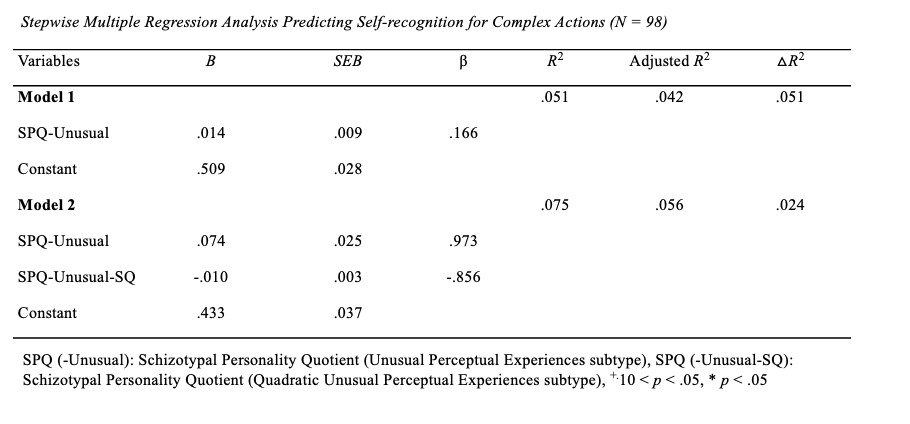


**Table S6.**
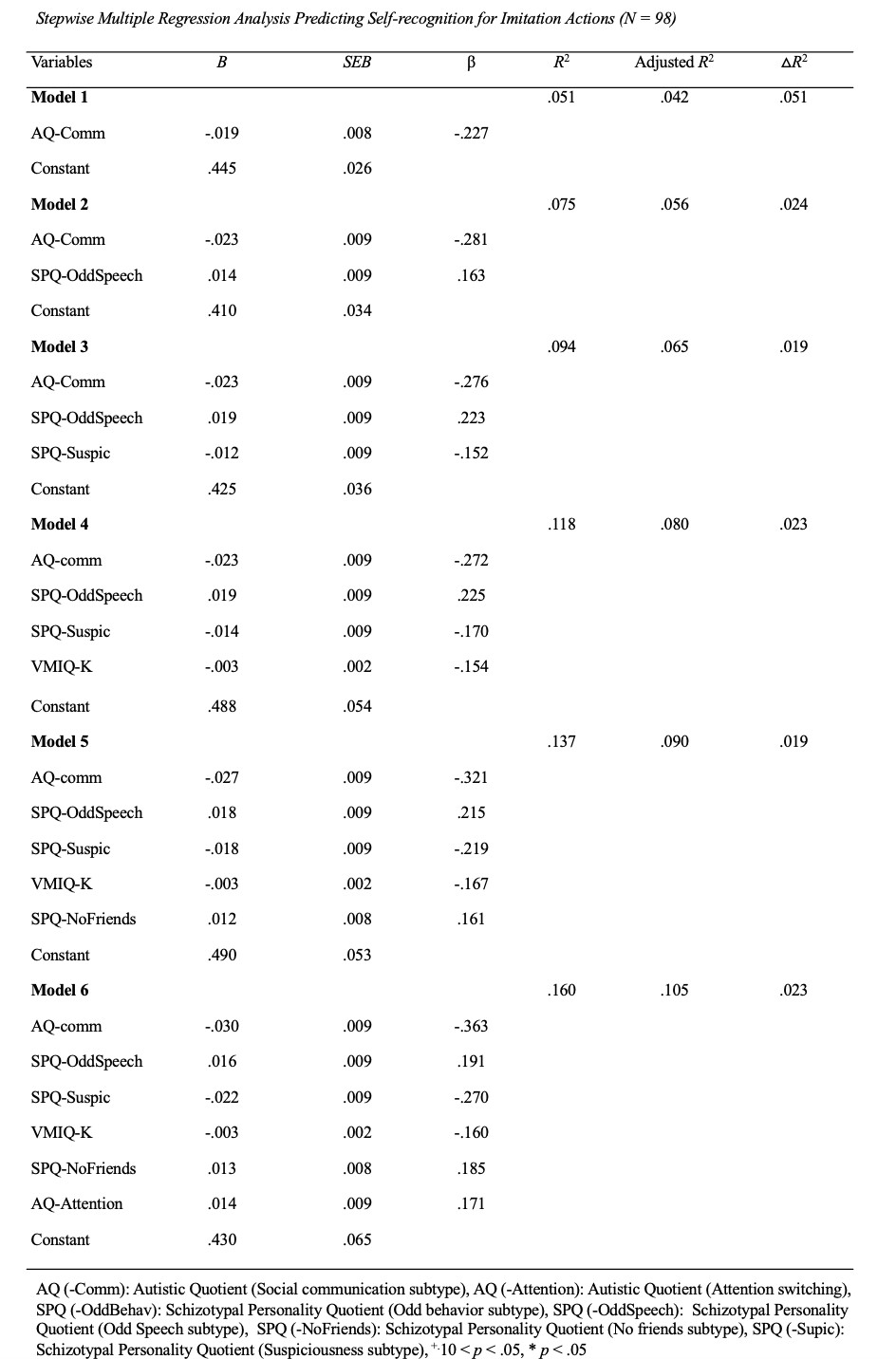


**Table S7.**


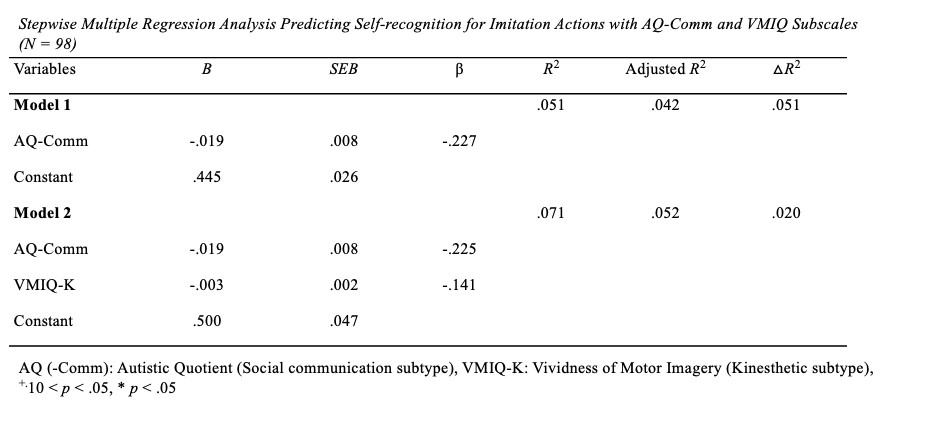


# CONFIDENCE DATA

# SVIII. Metacognitive assessments of self-recognition performance

Seventy-four participants provided confidence ratings for the self-recognition judgments. One-way repeated measures ANOVA revealed participants’ confidence ratings for self-recognition reflected performance accuracy based on action type, *F*(2,146) = 6.35, *p* = .002, η_p_^2^ = .080. Participants were significantly more confident for self-recognition for complex than for simple actions (*t*(73) = 4.05, *p* < .001, *d* = .471) and for imitation actions (*t*(73) = 2.04, *p* = .046, *d* = .237), while there was no difference in confidence judgments between imitation and simple actions (*t*(73) = 1.25, *p* = 0.217, *d* = .145).

**References**

1. Jokisch, D., Daum, I., & Troje, N. F. (2006). Self recognition versus recognition of others by biological motion: Viewpoint-dependent effects. *Perception*, *35*(7), 911-920.

1. Bates, D., Mächler, M., Bolker, B., & Walker, S. (2015). Fitting linear mixed-effects models using lme4. Journal of Statistical Software, 67(1), 1-48. https://doi.org/10.18637/jss.v067.i01

1. Kuznetsova, A., Brockhoff, P. B., & Christensen, R. H. (2017). lmerTest package: tests in linear mixed effects models. *Journal of statistical software*, *82*, 1-26.

1. Lenth, R., Singmann, H., Love, J., Buerkner, P., & Herve, M. (2021). Emmeans: Estimated marginal means, aka least-squares means. R Package Version 1 (2018).
